# Anaerobic glucose uptake in *Pseudomonas putida* KT2440 in a bioelectrochemical system

**DOI:** 10.1111/1751-7915.14375

**Published:** 2023-11-22

**Authors:** Laura Pause, Anna Weimer, Nicolas T. Wirth, Anh Vu Nguyen, Claudius Lenz, Michael Kohlstedt, Christoph Wittmann, Pablo I. Nikel, Bin Lai, Jens O. Krömer

**Affiliations:** ^1^ Systems Biotechnology group Helmholtz Centre for Environmental Research – UFZ Leipzig Germany; ^2^ Institute of Systems Biotechnology Saarland University Saarbrücken Germany; ^3^ Systems Environmental Microbiology Group, The Novo Nordisk Foundation Center for Biosustainability Technical University of Denmark Lyngby Denmark; ^4^ BMBF Junior Research Group Biophotovoltaics Helmholtz Centre for Environmental Research – UFZ Leipzig Germany

## Abstract

Providing an anodic potential in a bio‐electrochemical system to the obligate aerobe *Pseudomonas putida* enables anaerobic survival and allows the cells to overcome redox imbalances. In this setup, the bacteria could be exploited to produce chemicals via oxidative pathways at high yield. However, the absence of anaerobic growth and low carbon turnover rates remain as obstacles for the application of such an electro‐fermentation technology. Growth and carbon turnover start with carbon uptake into the periplasm and cytosol. *P. putida* KT2440 has three native transporting systems for glucose, each differing in energy and redox demand. This architecture previously led to the hypothesis that internal redox and energy constraints ultimately limit cytoplasmic carbon utilization in a bio‐electrochemical system. However, it remains largely unclear which uptake route is predominantly used by *P. putida* under electro‐fermentative conditions. To elucidate this, we created three gene deletion mutants of *P. putida* KT2440, forcing the cells to exclusively utilize one of the routes. When grown in a bio‐electrochemical system, the pathway mutants were heavily affected in terms of sugar consumption, current output and product formation. Surprisingly, however, we found that about half of the acetate formed in the cytoplasm originated from carbon that was put into the system via the inoculation biomass, while the other half came from the consumption of substrate. The deletion of individual sugar uptake routes did not alter significantly the secreted acetate concentrations among different strains even with different carbon sources. This means that the stoichiometry of the sugar uptake routes is not a limiting factor during electro‐fermentation and that the low rates might be caused by other reasons, for example energy limitations or a yet‐to‐be‐identified oxygen‐dependent regulatory mechanism.

## Graphical abstract

Providing an anodic potential in a bio‐electrochemical system to the obligate aerobe *Pseudomonas putida* enables anaerobic survival and allows the cells to overcome redox imbalances to achieve high yield of products. However, it is largely unclear how *P. putida* uptake the carbon into cytosol under electro‐fermentative conditions, while the cells have three native transporting systems for glucose differing in energy and redox demand. By creating three gene deletion mutants of *P. putida* KT2440 that force the cells to exclusively utilize one of the three routes, our results showed the gene deletion did not alter significantly the secreted acetate concentrations and thus suggested the stoichiometry of the sugar uptake routes was not a limiting factor during electro‐fermentation.

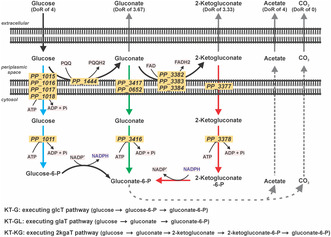

## INTRODUCTION

*Pseudomonas putida* is a promising host for industrial biotechnology (Martínez‐García & de Lorenzo, 2019; Nikel & de Lorenzo, 2018; Weimer et al., 2020). Due to its versatile central carbon metabolism and highly dynamic control of cellular energy and redox metabolism (Loeschcke & Thies, 2015; Nikel et al., 2015), this soil bacterium can withstand a range of stress conditions, such as extreme pH values, temperature gradients and high concentrations of toxic substances (Jimenez et al., 2002; Poblete‐Castro et al., 2012, 2020). These features inspired the engineering of *P. putida* into a microbial production host for chemicals (e.g. aromatics, acids and. alcohols) based on a variety of carbon substrates (Bitzenhofer et al., 2023; Dvořák & de Lorenzo, 2018; Loeschcke & Thies, 2020; Tiso et al., 2022; Wirth & Nikel, 2021). However, *P. putida* relies solely on oxygen as the terminal electron acceptor. On the one hand, this lifestyle allows *P. putida* to generate abundant energy facilitating high metabolic turnover rates while being supplied with oxygen. However, this situation also limits scalability, increases process costs and leads to carbon loss due to full oxidation of substrates, all common problems of aerobic bioprocesses (Hannon et al., 2007). Consequently, developing an anaerobic mutant strain of *P. putida* has been a long‐term desire for improving its economic feasibility in industrial applications.

Different efforts have been made to implement fermentative pathways towards ethanol and acetate (Nikel & de Lorenzo, 2013) or establish anaerobic nitrate respiration (Steen et al., 2013) in *P. putida*, but with limited success. Recently, *in‐silico* simulations addressed the issue of oxygen dependence by predicting oxygen‐dependent essential metabolic functions for growth, followed by their replacement with oxygen‐independent alternatives (Kampers et al., 2019). The resulting strain of *P. putida* KT2440 achieved extended survival and restricted metabolic activities under micro‐oxic conditions but was still unable to survive under anoxic conditions (Kampers et al., 2021). Further strain development is needed and additional metabolic constraints remain to be identified.

In contrast to metabolic engineering strategies, an alternative approach to balance the cellular energy and redox metabolism of *P. putida* in the absence of oxygen is based on bio‐electrochemical systems (BES) that offer extracellular electron sinks (Askitosari et al., 2019, 2020; Chukwubuikem et al., 2021; Hintermayer et al., 2016; Lai, Yu, et al., 2016; Schmitz et al., 2015). These studies have found that a redox mediator is necessary to withdraw electrons from the membrane‐bound electron transfer chain, acting as a cellular electron acceptor and constantly being re‐oxidized by the anode. This makes this process inexhaustible compared to the formation of by‐products or the consumption of co‐substrates often required for redox balancing in fermentations (except for some cases, e.g. lactate fermentation from glucose [Shukla et al., 2004]). *P. putida* was shown to actively drive glucose metabolism in such an oxygen‐free environment (Chukwubuikem et al., 2021; Lai et al., 2020) with 2‐ketogluconic acid as the main end‐product with very high yields of above 90%. Furthermore, it was shown in previous studies, that *P. putida* was able to metabolize a wide range of aldoses and fructose in BES, like. Therefore, the so far known possible product spectrum also includes (keto‐)aldonic acids, like (2‐keto) galactonic acid and arabinonic acid (Nguyen et al., 2021). This process might be also transferred to other compounds that could benefit from the anodic fermentation process (Kracke & Krömer, 2014) if the metabolic constrains of carbon turnover and uptake could be solved in the future.

While this BES system allowed the consumption of glucose, it remains unclear how the cells performed cytosolic glucose oxidation in the BES and anaerobic growth (i.e. biomass formation) could not be observed. Glucose was mainly redirected towards periplasmic oxidation with 2‐ketogluconate as the main end product (Lai et al.,  2020; Lai, Yu, et al., 2016). Only about 10% of the carbon was catabolized through the cytosolic central carbon metabolism, resulting in the production of acetate as the sole reliably detectable by‐product. When cultured in the presence of the anode (Lai, Yu, et al., 2016), *P. putida* was found to achieve a comparable adenylate energy charge in the BES compared to aerobic cultures, despite much lower absolute intracellular ATP levels. This indicated that energy metabolism at the kinetic level is limited in the flow of electrons between the cells and the electrode, and thus cannot meet the high demands required for growth. However, the intracellular redox NAD^+^/NADH and NADP^+^/NADPH ratios were shifted to the oxidized states, indicating that the BES was withdrawing electrons faster than central metabolism could replenish them, which might demonstrate that sugar uptake and catabolism is too slow.

**FIGURE 1:**
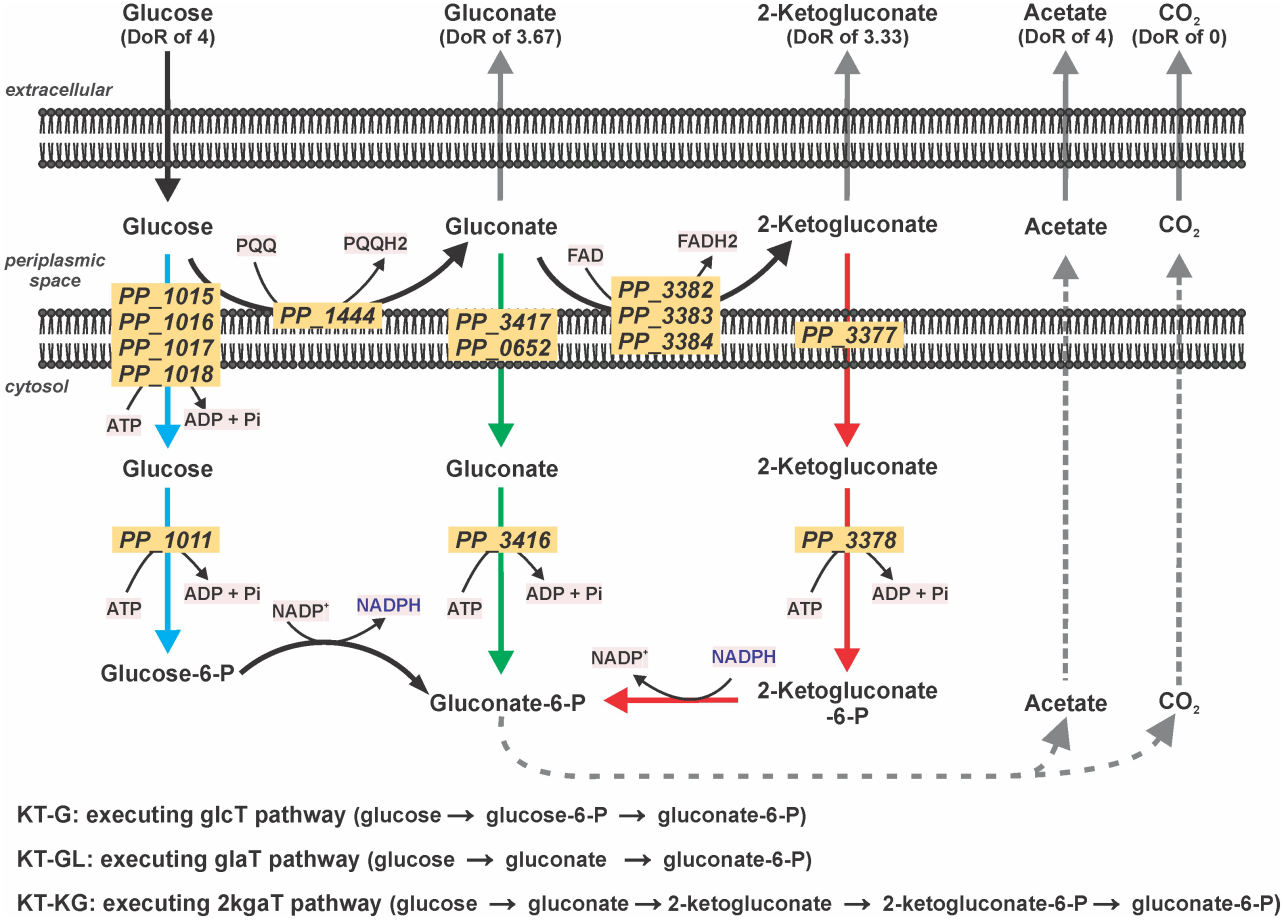
Glucose oxidation pathways of *Pseudomonas putida* in BES. Three pathways exist to convert extracellular glucose to intracellular gluconate‐6‐P (which is further converted to acetate): Left (blue): via glucose (**glcT** pathway); Centre (green): via gluconate (**glaT** pathway); Right (red): via 2‐ketogluconate (**2kgaT** pathway). The three deletion mutants **KT‐G**, **KT‐GL** and **KT‐KG** were engineered to each use only one of the three entry routes. Each pathway affects the NADP^+^/NADPH ratio differently also the ATP demands. DoR, degree of reduction.

To elucidate the underlying processes and provide a target for the development of a more active phenotype through metabolic engineering, the pathway of sugar uptake under BES conditions represented a major knowledge gap. *P. putida* has three potential pathways to convert glucose into gluconate‐6‐phosphate (the common entry point into cytosolic central metabolism). Each pathway results in a distinct NADPH yield (Figure 1). Under aerobic conditions, *P. putida* mainly takes up glucose via a gluconate transporter (**glaT** pathway) (Kohlstedt & Wittmann, 2019; Nikel et al., 2015), which leads to a reduction of pyrroloquinoline quinone (PQQ), but without affecting the NADPH pool directly. Carbon uptake via 2‐ketogluconate (**2kgaT** pathway) generates four electrons (PQQH_2_ and FADH_2_) but oxidizes one NADPH. The uptake via a glucose ABC transporter (**glcT** pathway) does not lead to periplasmic oxidation but requires one NADP^+^ (or 2/3 NAD^+^ and 1/3 NADP^+^ proposed by Olavarria et al. (2015)). The three pathways also pose different demands on ATP hydrolysis. The **glcT** pathway was well‐studied with outer membrane porin (Saravolac et al., 1991) and inner membrane ABC transporter (Pandey et al., 2016) being involved, and requires two ATPs from glucose to reach the gluconate‐6‐phosphate. In contrast, the biochemical mechanisms of the gluconate and 2‐ketogluconate transporters in *P. putida* are not yet understood; but, it is generally accepted that they do not belong to the ABC transporter family and do not require the hydrolysis of ATP for their biochemical function (del Castillo et al., 2007). Therefore, both **glaT** and **2kgaT** pathways only require one ATP to convert glucose to gluconate‐6‐phosphate.

In this work, we studied the prevalent carbon uptake across the cytoplasmic membrane in *P. putida* KT2440 during anaerobic cultivation in a BES reactor. A set of deletion mutants was constructed and the activity of glucose oxidation was studied through generated current, the formation of extracellular metabolites, the metabolization of ^13^C‐glucose as an isotopic tracer, and metabolic and flux balance analysis. The obtained results excluded limitations in redox balancing as the limiting factor for glucose uptake but pointed to a potential regulatory mechanism sensitive to oxygen, which remains to be identified.

## EXPERIMENTAL PROCEDURES

### Strains

The *P. putida* strains used in this study are listed and explained in Table 1 below.

**TABLE 1:** Strains created and used in this study.

*P*. *putida* strains	Genotype	Description	Source
KT2440	Wild‐type strain	Derivative of strain mt‐2, cured from the pWWO catabolic plasmid	Bagdasarian et al. (1981)
KT‐G	*P. putida* KT2440 Δ*gcd*	In‐frame gene deletion mutant of glucose dehydrogenase (*gcd*, *PP_1444*); streamlined for cross‐membrane transportation pathway via glucose	Sánchez‐Pascuala et al. (2019)
KT‐GL	*P. putida* KT2440 Δ*glk* Δ*gtsABCD* Δ*gad*	In‐frame gene deletion mutant of glucose ABC transporter (*gtsABCD*, *PP_1015*‐*PP_1018*), glucokinase (*glk*, *PP_1011*) and gluconate dehydrogenase (*gad*, *PP_3382*‐*PP_3384*); streamlined for cross‐membrane transportation pathway via gluconate	This study
KT‐KG	*P. putida* KT2440 Δ*glk* Δ*gtsABCD* Δ*gnuK* Δ*gntT* Δ*PP_0652*	In‐frame gene deletion mutant of glucose ABC transporter (*gtsABCD*, *PP_1015*‐*PP_1018*), glucokinase (*glk*, *PP_1011*), gluconate transporters (*gntT*, *PP_3417* and *PP_0652*) and gluconokinase (*gnuK*, *PP_3416*); streamlined for cross‐membrane transportation pathway via 2‐ketogluconate	This study

Genetic deletions in *P. putida* were carried out following previously published protocols (Volke et al., 2021). In brief, homology arms (HA) flanking the chromosomal sequences targeted for deletion of about 500 bp were cloned into the suicide vector pSNW2 using Uracil‐excision (USER) cloning (Cavaleiro et al., 2015). *Escherichia coli* DH5α λpir served as the cloning host. Chemically‐competent *E. coli* cells were prepared and transformed with plasmids according to well‐established methods (Calero et al., 2020). The specific pSNW2 plasmids were introduced into *Pseudomonas* via electroporation (Choi et al., 2006) and integrated into the target locus through homologous recombination. Subsequently, cells were transformed with a second plasmid, pQURE6, which encodes the meganuclease I‐*Sce*I. This enzyme introduces double‐strand breaks within the backbone of the chromosomal pSNW2, forcing a second homologous recombination event that results in the resolution of the suicide vector and potentially yielding the desired genetic modification. Oligonucleotides used to amplify HAs are listed in Supplementary Table S1. After cloning procedures and genome manipulations, colony PCRs were conducted for genotyping experiments using the commercial OneTaq™ master mix (New England BioLabs; Ipswich, MA, USA) as per the manufacturer's instructions.

### Growth medium and conditions

A defined M9 medium (DM9) with glucose as the sole substrate was used for the cultivation of *P. putida* cells. The detailed recipe can be found elsewhere (Lai, Yu, et al., 2016). To minimize systematic errors, each batch of experiments was started from cryo‐stocks of the respective strains by streaking on an LB agar plate and incubating for about 24 h at 30°C (Heratherm™ Microbiogical Incubator, Thermo Scientific). Afterwards, a single colony was picked from the LB plate and transferred into a baffled shake flask containing DM9 medium for inoculation. A maximum of 20% of the working volume of the flasks was filled with medium for all growth experiments in this work. The liquid culture was incubated at 30°C and 200 rpm (Multitron Pro, INFORS HT) until the desired cell density was reached, which was measured using a spectrophotometer (Libra S12, Biochrom Ltd, Cambridge, Great Britain; DM9 medium as blank) at the wavelength of 600 nm (OD600). The cell dry weight (CDW) was then calculated using the following formula: CDW [g/L] = 0.486 * OD600 (Yu et al., 2018). The correlation factor was assumed to be the same for all strains used in this study.

For the inoculation of the BES reactors, the cells were harvested at the end of the exponential growth phase at the OD_600_ of ~3. All BES reactors were started with an initial OD600 of ~1.0. For this purpose, a respective preculture volume was centrifuged at 7000 *g*, 25°C for 10 min, resuspended in fresh DM9 media without glucose, and then injected into the BES reactor using a syringe.

### Aerobic growth kinetics determination

To determine the lag phase of the growth, the cryo stocks were activated on the LB agar plate for 24‐36 h. Afterwards, colonies were picked using an inoculation loop and then inoculated into the DM9 liquid medium with 5 g/L glucose. Samples were then taken in regular intervals to monitor the growth at OD_600_.

The exponential growth phase kinetics were determined by preparing the DM9 liquid precultures from LB plates. Fresh DM9 medium was inoculated with the four strains to reach an initial OD_600_ of ~0.1, and then the growth profiles were monitored over the day time. The exponential growth phase was defined by the linear plot of LN(OD_600_) against the time, while the maximum growth rates (μ, h^−1^) were determined by the slopes. The biomass yield of the exponential growth phase (*Y*_*x*/*s*_, [*g*_CDW_/*g*_glucose_]*)* was determined by the slope of linear fitting between the biomass density (in cell dry weight, [*g*_CDW_/L]) measured by OD600 versus the glucose concentrations ([g/L]) measured by HPLC. Finally, the glucose consumption rate (*r*_glucose_, [mmol_glucose_/g_CDW_/h]) was calculated by the following equation: *r*_glucose_ [mmol_glucose_/*g*_CDW_/h] = μ [h^−1^] / (Y_x/s_ [*g*_CDW_/*g*_glucose_] * MW_glucose_ [g/mmol], while MW_glucose_ is the molecular weight of glucose (0.18016 g/mmol).

### Bioelectrochemical system setup

The setup and parameters of the bioelectrochemical system were described in detail elsewhere (Lai et al., 2019). Briefly, for the BES fermentations a three‐electrode system was used. Carbon cloth (25 cm^2^, 1071HCB, FuelCellStore, Texas, USA) pretreated with 2 mM cetrimonium bromide to improve its surface hydrophilicity was supplied as anode, and stainless‐steel mesh (FE621018, Advent Research Materials, Oxford, England) was used as cathode. An Ag/AgCl electrode in saturated KCl (RE‐1CP, Als, Tokyo, Japan, Cat. No.: 013691) was deployed as reference electrode. The working electrode potential was set to 0.697 V vs standard hydrogen electrode (SHE) for all BES measurements, if applicable. DM9 medium with 1.5 g/L glucose (^13^C_6_‐glucose or ^12^C_6_‐glucose) as the sole carbon source was used as working medium. For the isotope tracer experiments in BES, precultures were grown in media with naturally labelled glucose as substrate and then inoculated into BES reactors containing ^13^C_6_‐glucose. Ferricyanide was added to the working chamber to a final concentration of 1 mM as electron transfer mediator. Under our experimental conditions ferricyanide is a stable complex. The combined total amount of ferro‐ and ferricyanide was constant. This was quantified using a colorimetric method of measuring the optical density of the supernatant at 420 and 320 nm respectively (Lai, Yu, et al., 2016). The working chamber was kept at 30°C, flushed with N_2_ gas (~20 mL/min) in the headspace and mixed via magnetic stirring at 400 rpm.

### Measurement of exo‐metabolites

The supernatants from the shake flasks and BES reactors were obtained by centrifuging the liquid samples at 4°C and 17,000 *g* for 10 min. The obtained supernatants were then stored at −20°C until further analysis. High‐performance liquid chromatography (UltiMate 3000, Thermo Fisher Scientific, Massachusetts, USA) with Hi‐Plex H column (PL1170‐6830, 300 × 7.7 mm, Agilent Technologies) was used to detect and quantify glucose and organic acids (including gluconate, 2‐ketogluconate, acetate, succinate, pyruvate, formate, etc.). The HPLC method used is described in detail elsewhere (Lai, Plan, et al., 2016; Lai, Yu, et al., 2016). Briefly, the organic acids were analysed with the following conditions: column temperature of 40°C, mobile phase 14 mM H_2_SO_4_ at 0.4 mL/min. For sugars, the running parameters were: column temperature of 15°C, mobile phase milliQ H_2_O at 0.4 mL/min.

### Isotopic labelling measurements

The presence of acetate as well as its isotopomer distribution was verified and determined using ion chromatography (Dionex™ ICS‐6000 Capillary HPIC™ System, Thermo Fischer) coupled to a mass spectrometer (Orbitrap Exploris™ 240, Thermo Fischer) (IC‐MS). To prepare the samples for IC‐MS, the supernatants were desalted using butanol extraction. For this purpose, the pH of the samples was decreased to 1–2 by the addition of H_2_SO_4_. Subsequently, 1‐butanol was added in a ratio of 1:1 and mixed thoroughly. While the acetate is extracted to the organic phase, the salts will remain in the aqueous phase, hence the butanol phase was used for the IC‐MS measurement. Afterwards, 20% of methanol and KOH (final concentration of 1 mM) was added to the desalted samples. For ion chromatography, a Dionex™ IonPac™ AS11‐HC IC column was used. The eluent flow was adjusted to 0.38 mL/min and the injection volume was set to 10 μL. The hydroxy peroxide concentration of the eluent was gradually increased from 1 to 60 mM over the course of 33 min for elution plus 10 min for equilibration. The regenerant pump was set to 0.5 mL/min and via a make‐up pump methanol was spiked into the flow with 0.15 mL/min to improve the ESI efficiency. The MS was operated in targeted data‐dependent MS2 mode in the range of 40–200 m/z with a vaporizer temperature of 300°C and ion transfer tuber temperature of 325°C.

The mass isotopomer distribution of secreted acetate was also determined using a gas chromatograph coupled to mass spectrometry (GC–MS) (Agilent 7890A, Quadrupole Mass Selective Detector 5975C, Agilent Technologies, Santa Clara, California, USA). The instrument was equipped with an HP‐5MS column as stationary phase (30 m, 250 × 0.25 μm, Agilent Technologies). Helium (5.0) was used as the mobile phase. Prior to analysis, acetate was derivatized with n‐pentanol, followed by extraction with n‐hexane (Adler et al., 2013, 2014). For this means, 50 μL of culture supernatant was mixed with 100 μL H_2_SO_4_ (10% vol/vol) and 20 μL n‐pentanol, incubated at 80°C for 15 min, subsequently cooled down to 5°C and extracted with 200 μL n‐hexane. The oven program was as follows: 75°C for 2 min, ramp: 25°C min^−1^, final temperature: 300°C). Samples were analysed in selected ion monitoring (SIM) mode at *m/z* 43 to 45 to obtain the mass isotopomer fractions m + 0, m + 1 and m + 2 of a fragment ion that contained both carbon atoms of acetate. For method validation, 0.5% (w/vol) solutions of naturally labelled sodium acetate and [^13^C_2_] acetic acid (99% isotopic purity, Cambridge Isotopes) were treated as described above and the respective ion clusters were evaluated.

The mass isotopomer distribution of pyruvate was determined using GC–MS (Kiefer et al., 2002). Briefly, 200 μL of fermentation supernatant was dried under a nitrogen stream. Derivatization was done in a two‐step procedure: First, dried samples were dissolved in 50 μL methoxyamine hydrochloride in pyridine (20 mg/mL), and incubated at 80°C for 30 min. Afterwards, 50 μL MSTFA (Macherey‐Nagel, Düren, Germany) were added, and incubated likewise. Mass fragment distribution of pyruvate was determined by GC/MS (Agilent 7890A, Quadrupole Mass Selective Detector 5975C, Agilent Technologies) using the following oven program: 30°C (0–1 min), 10°C min^−1^ increase (1–10 min) and 40°C min^−1^ increase (10–15.125 min). Selected ion monitoring (SIM) targeting m/z 174 was performed to quantify the mass isotopomer fractions *m + 0*, *m + 1*, *m + 2*, *m + 3* of a fragment ion containing all carbon atoms of pyruvate. The obtained ^13^C enrichment was corrected for the natural abundance of ^13^C (van Winden et al., 2002) and was expressed as summed fractional labelling (SFL) (Wittmann & Heinzle, 2005).

### Flux balance analysis

The flux balance analysis was conducted based on the product yields on glucose and glucose consumption rates over the BES fermentation. The product yields were calculated by plotting the product (i.e. gluconate, 2‐ketogluconate, acetate and pyruvate) concentration versus glucose concentration over the BES batches, where the slope of the linear fitting indicated the value of the yield. Due to the dynamic biomass change over the BES batch, the specific glucose consumption rate (*r*_*s*_, [mmol/g_CDW_/h]) was calculated in two steps: (1) the rate in the unit of [mmol/L/h] was calculated by plotting the glucose concentration versus time over the batch; (2) the rate value was then further divided by an integral averaged biomass density ([g_CDW_/L]) to reach the unit of [mmol/g_CDW_/h]. The flux of the product was then calculated using the formula: *r*_*p*_ = *r*_*s*_ * *Y*_*p*/*s*_.

## RESULTS

Three strains of *P. putida* KT440 were constructed, which could import glucose exclusively through only one of the distinct pathways while the other two were disabled through respective gene deletions. These strains were named KT‐G, KT‐GL and KT‐KG, corresponding to the glcT, glaT and 2kgaT pathways, respectively (Figure 1, Table 1).

### Effect of gene deletion of sugar kinases and membrane transporters on aerobic growth

**FIGURE 2:**
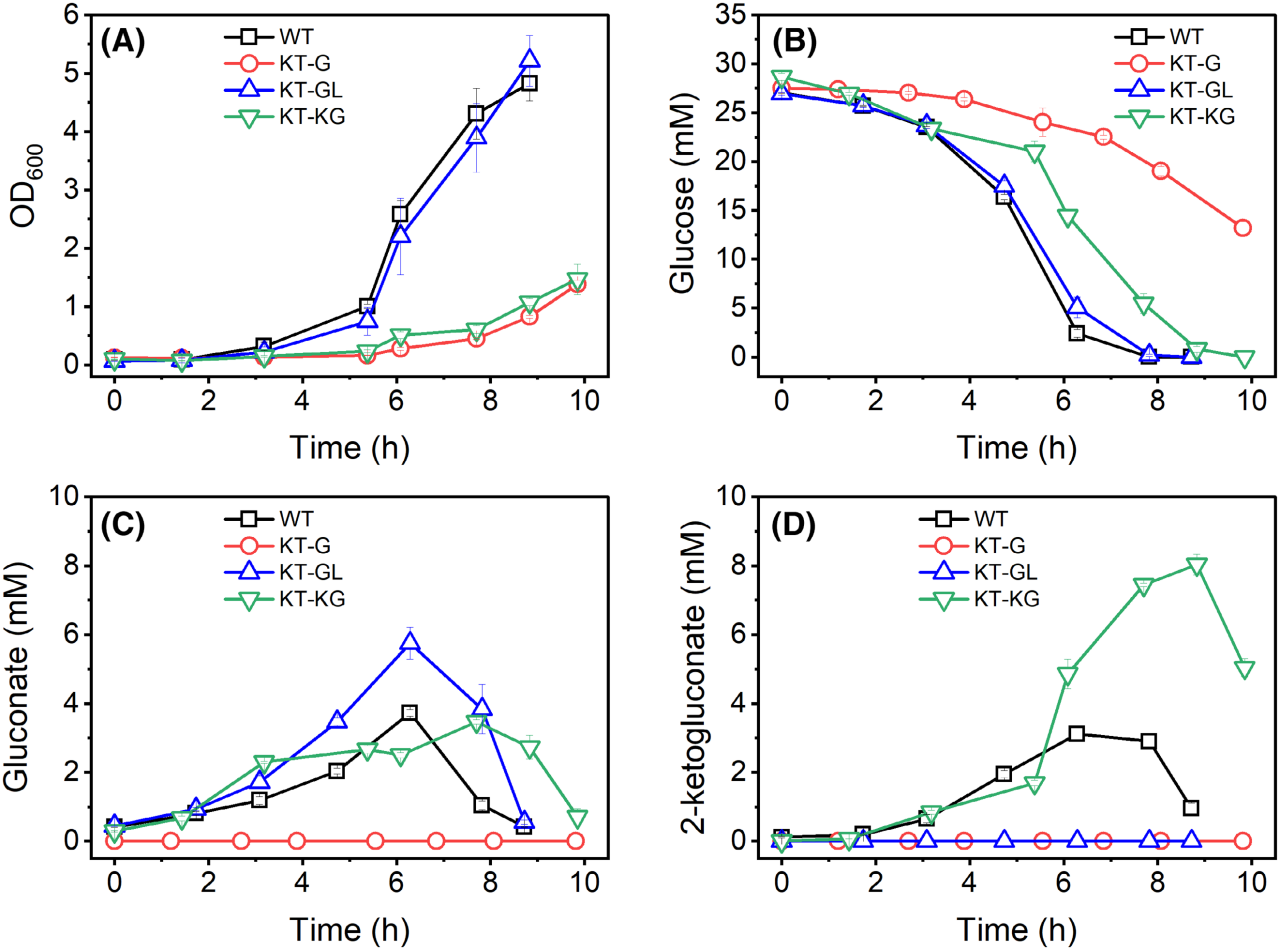
Cultivation of *P. putida* KT2440 WT and three gene deletion mutants in minimal medium with glucose as substrate under aerobic conditions. (A) Growth of all four strains measured via OD_600_. (B–D) Extracellular concentrations of glucose, gluconate and 2‐ketogluconate. The presented data are averages and standard deviations of 3 biological replicates. All cultures were inoculated to an OD_600_ of about 0.1.

In addition to molecular confirmation (e.g. by PCR of the cognate loci), we could confirm the phenotypic nature of the respective gene deletions based on aerobic extracellular metabolite profiles (Figure 2). The wild type consumed glucose and produced gluconate and 2‐ketogluconate as intermediate products during glucose‐dependent growth. The secretion of gluconate/2‐ketogluconate was completely eliminated for the KT‐G strain, while 2‐ketogluconate secretion was not detected for the KT‐GL mutant. The KT‐KG strain produced both intermediates but with higher 2‐ketogluconate amounts compared to the wild‐type strain.

The deletion of genes can lead to adverse effects on microbial growth and physiology (Takeuchi et al., 2014). Therefore, we further determined the aerobic growth kinetics of the recombinant strains compared to the parental strain KT2440 (WT) (Figure 2 and Table 2). The mutant KT‐GL exhibited a similar growth profile compared to the WT. In contrast, the growth of the strains KT‐G and KT‐KG was significantly impaired, with maximum growth rates only reaching about 50% of the WT. Additionally, forcing the utilization of the 2kgaT pathway (i.e. KT‐KG strain) resulted in a longer lag phase when cells were initially transferred from solid agar to liquid cultures (see Supplementary Figure S1). Once the cells adapted to glucose conditions, this lag phase was eliminated in subsequent cultivations in liquid medium (Table 2).

**TABLE 2:** Growth kinetics of wild‐type *P. putida* KT2440 and the respective gene deletion mutants in minimal medium with glucose as substrate under aerobic conditions.

*P. putida* strains	*μ*_max_ [h^−1^]	*r*_glucose_ [mmol/*g*_CDW_/h]	*Y*_*x*/*s*_ [*g*_CDW_/*g*_glucose_]	*r_glucose_ [mmol/*g*_CDW_/h]	*Y_*x*/*s*_ [*g*_CDW_/*g*_glucose_]
KT2440 WT	0.74 ± 0.05	−14.17 ± 1.48	0.29 ± 0.02	−10.69 ± 0.95	0.38 ± 0.02
KT‐G	0.37 ± 0.01	−7.99 ± 0.58	0.26 ± 0.02	−7.99 ± 0.58	0.26 ± 0.02
KT‐GL	0.75 ± 0.10	−14.91 ± 4.40	0.28 ± 0.07	−11.56 ± 3.08	0.36 ± 0.08
KT‐KG	0.35 ± 0.01	−16.77 ± 1.35	0.12 ± 0.01	−11.92 ± 1.18	0.16 ± 0.02

*Note*: Rates and yields were calculated for the exponential growth phase from 3 biological replicates. The exponential growth phases for the WT, KT‐G, KT‐GL and KT‐KG were defined as 1.7–6.3 h (*R* ^2^ = 0.991), 3.9–9.8 h (*R* ^2^ = 0.993), 1.7–6.3 h (*R* ^2^ = 0.999) and 1.4–9.9 h (*R* ^2^ = 0.976) respectively (Supplementary Figure S2), corresponding to the timeline in Figure 1. The rate and yield values marked by * were calculated by excluding the periplasmic oxidative products (gluconate and 2‐ketogluconate) from the calculations, thus indicating the net glucose being taken up into the cytosol during the exponential growth phases being examined. The growth curves determined using the Bio‐Lector online cultivation were provided in Supplementary Figure S3. *r*_glucose_, specific glucose uptake rate; *Y*_*x*/*s*_, biomass/glucose yield.

The gene deletions affected not only the growth rate but also the glucose consumption rate. Similar to the growth rate, KT‐GL neither differed from the WT in the specific glucose consumption rate nor in the biomass yield (Table 2). But despite the lower growth rate, KT‐KG consumed glucose at a similar rate as the WT, especially when comparing the net glucose consumption after excluding the periplasmic intermediate products (Table 2). Despite the similar glucose uptake rate, the biomass yield for KT‐KG was less than half of that for the WT, corresponding to the pattern of maximum growth rate. The opposite phenomenon was observed for the KT‐G strain. The glucose consumption rate was decreased by ~50% after the deletion of the glucose dehydrogenase gene, but the biomass yield remained at a comparable level to the WT.

### Electrogenic activity of *P. putida* mutants during BES fermentations

**FIGURE 3:**
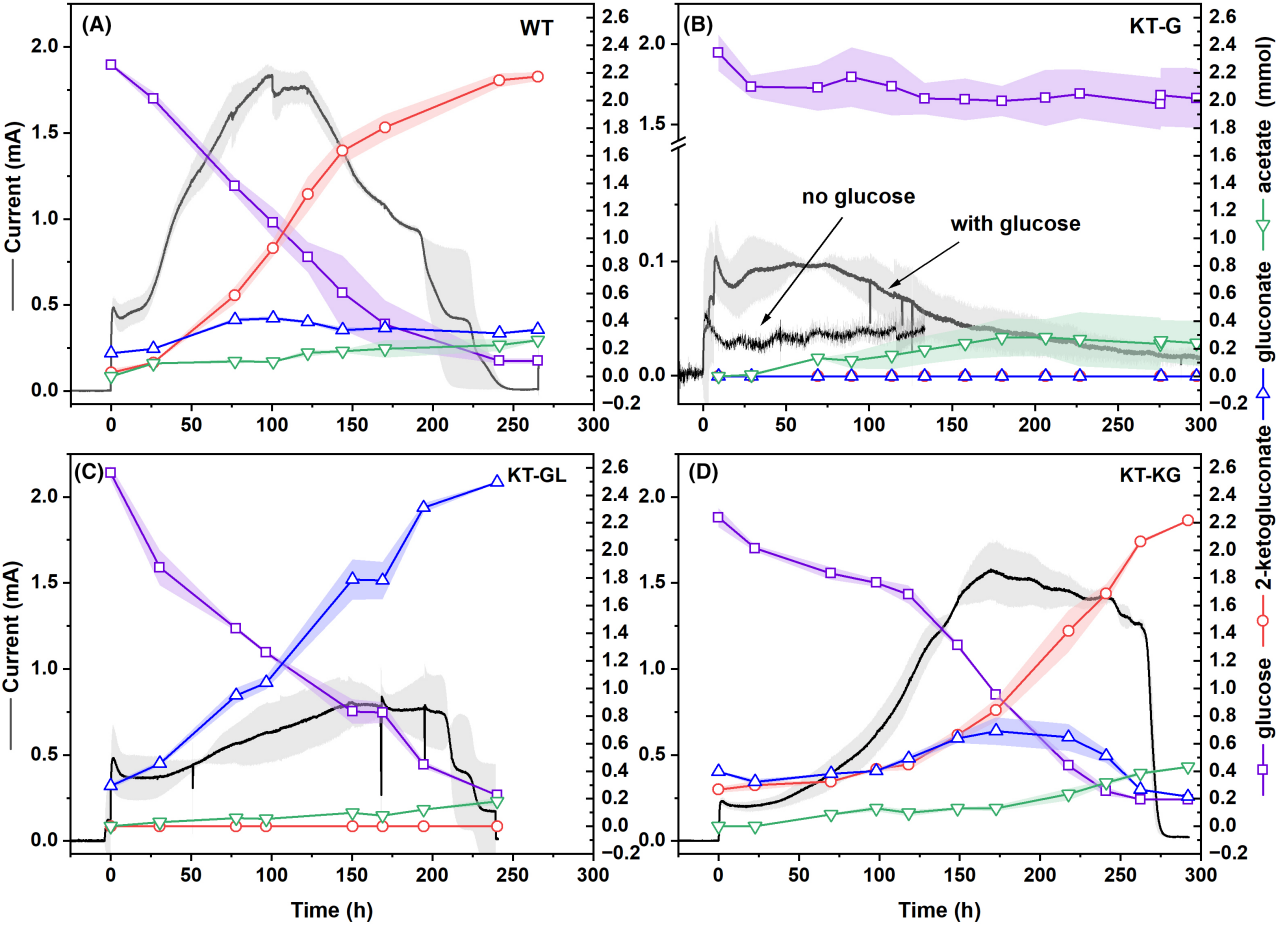
Representative performance of *P. putida* KT2440 WT and the three gene deletion mutants in BES. Left *y*‐axis indicates the current output of the respective BES fermentations, and the right *y*‐axis shows the quantity of metabolites (mmol) in the reactors. Due to the low sugar consumption by the KT‐G mutant, a control experiment without glucose was conducted to check the current response from carbon carry‐over originating from the preculture. The line or line‐symbol presents the averaged values and the shading indicates the standard deviations of biological replicates.

The electrogenic activity and the corresponding glucose metabolism of the WT and the three recombined strains were tested in the BES (Figure 3). The mutant KT‐KG showed a similar pattern to the WT, in terms of current density and metabolic products. The peak current reached 1.56 ± 0.22 mA, slightly higher than that of WT; but it took KT‐KG about 200 h after inoculation, compared to ~120 h for WT. Glucose was consumed slower than in the WT at a rate of 0.08 ± 0.01 mmol/g_CDW_/h (Table 3), but the product spectrum was comparable, resulting mainly in glucose conversion to 2‐ketogluconate.

**TABLE 3:** Yields and rates of *P. putida* KT2440 and the respective gene deletion mutants in BES.

*P. putida* strains	*Y*_mmol_acetate/mmol_glucose_	*Y*_mmol_electrons/mmol_glucose_	*r*_glucose_ [mmol/*g*_CDW_/h]	*r*_electrons_ [mmol/*g*_CDW_/h]
KT2440 WT	0.07 ± 0.01	4.37 ± 0.08	−0.19 ± 0.02	0.51 ± 0.10
KT‐G	0.48 ± 0.06	0.17 ± 0.13	−0.01 ± 0.01	0.002 ± 0.002
KT‐GL	0.07 ± 0.01	2.44 ± 0.12	−0.18 ± 0.01	0.45 ± 0.01
KT‐KG	0.18 ± 0.01	4.69 ± 0.10	−0.08 ± 0.01	0.39 ± 0.03

*Note*: The standard deviations were calculated from at least four biological replicates (WT: 6 replicates with 55 data points; KT‐G: 6 replicates with 51 data points; KT‐GL: 4 replicates with 26 data points; KT‐KG: 6 replicates with 56 data points). More kinetics and their calculations can be found in the Supplementary Table S2 and Supplementary Figures S4–S7.

In contrast, the strain KT‐GL produced a lower current output with a peak of 0.80 ± 0.15 mA at ~150 h after inoculation (Figure 3), compared to the WT. Despite the lower current output, KT‐GL consumed glucose at a rate (0.18 ± 0.00 mmol/g_CDW_/h) that was about 55% faster than WT, with gluconate as the main end‐product. In all, KT‐GL was able to reach an extracellular electron transfer rate of 0.45 ± 0.01 mmol_electrons_/g_CDW_/h, only about 13% decrease from the value 0.51 ± 0.10 mmol_electrons_/g_CDW_/h for the WT (Table 3). Regarding metabolic products from cytosolic metabolism, acetate was secreted as the only by‐product of both KT‐GL and KT‐KG mutants. The molar acetate yield of KT‐GL was the same as that of the WT (both 7.1 ± 1.1%), while it was higher for KT‐KG (17.5 ± 0.9%) (see Supplementary Table S2). The carbon and electron balances for the strains that consumed significant amounts of glucose could be closed for each biological replicate and reached 100% and 101.1% for the WT, 101.3% and 102.2% for KT‐GL, as well as 103.9% and 104.8% for KT‐KG, respectively (see Supplementary Table S2).

The current output measured for the strain KT‐G was significantly lower compared to the other two mutants and the WT, with a peak current of only 0.09 ± 0.03 mA (Figure 3). The current output also only lasted for a short period. Furthermore, only about 0.3 mmol glucose was consumed by the KT‐G mutant over 300 h of cultivation in BES, and the average consumption rate was only ~0.01 mmol/g_CDW_/h (Table 3). No gluconate and 2‐ketogluconate were detected; however, a similar amount of acetate was measured for KT‐G as for the other strains (Supplementary Figure S8). In a control experiment without added glucose there was also an observable current response (peak at around 0.05 mA) (Figure 3). This indicates that current output could also be generated from intracellular metabolism when acetate formation is observed. A very low amount of pyruvate was measured in HPLC (Supplementary Figure S5) and further confirmed by GC–MS with isotopic tracer analysis (Supplementary Figure S9). A similarly low current output was also observed for the KT‐GL strain while using gluconate as the sole substrate in the BES system. Only about 0.35 mmol gluconate was consumed and a similar amount of acetate to the glucose cases in BES was measured (Supplementary Figures S8 and S10). Isotopic enrichment analysis also confirmed about 64% of the carbon in acetate came from the gluconate in the BES (Supplementary Figure S10).

### ^13^C enrichment in extracellular acetate during BES fermentation

**FIGURE 4:**
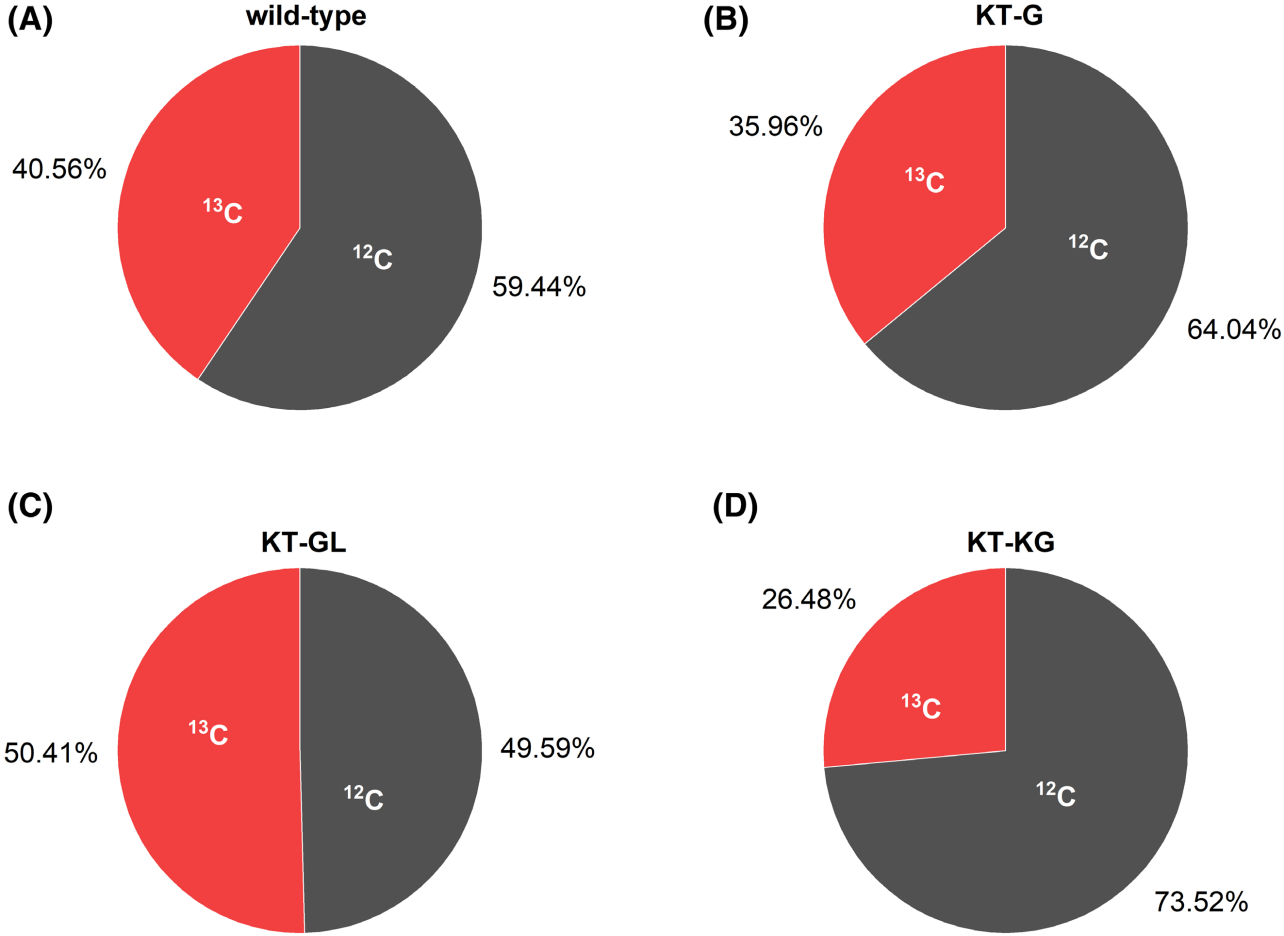
The summed fractional labelling (SFL) of ^13^C in endogenous acetate for the wild type and recombinant strains in BES with ^13^C_6_‐glucose as the substrate. The isotope enrichment was detected using both GC–MS and IC‐MS on samples from three independent biological replicates. Colour code: red, the ^13^C originated from the de novo synthesis from the fed‐in ^13^C_6_‐glucose; dark grey, the ^12^C originated from other sources.

Acetate was the only significant by‐product in all fermentations not related to the periplasmic oxidation pathway. We observed only small amounts of acetate after the BES fermentations. An increase in acetate formation was observed over the batch when glucose was provided, but also a trace amount of acetate was detected in the BES controls without glucose addition (Supplementary Figure S8). To identify the origin of the carbon and to determine the ratio between glucose‐derived acetate and acetate from other sources, we provided ^13^C_6_‐glucose as a carbon source and analysed the ^13^C enrichment in extracellular acetate using mass spectrometry (Figure 4).

For all strains, ^13^C labelling was detected in acetate. After correcting for naturally occurring isotopes in the analytes, the summed fractional labelling (SFL) for carbon was calculated. The determined SFL values indicated a ^13^C enrichment in acetate between ~26% for the KT‐KG strain and about 50% for the KT‐GL strain. This means that half of the carbon in the acetate could be traced back to the substrate ^13^C_6_‐glucose in the BES reactor in one case, while in other cases the share was well below 50% and carbon sources originating from the initial biomass inoculum (e.g. products of cell lysis in long BES experiments or usage of intracellular storage compounds, such as PHA, etc) being the major contributor to acetate formation.

### Flux balance analysis

**FIGURE 5:**
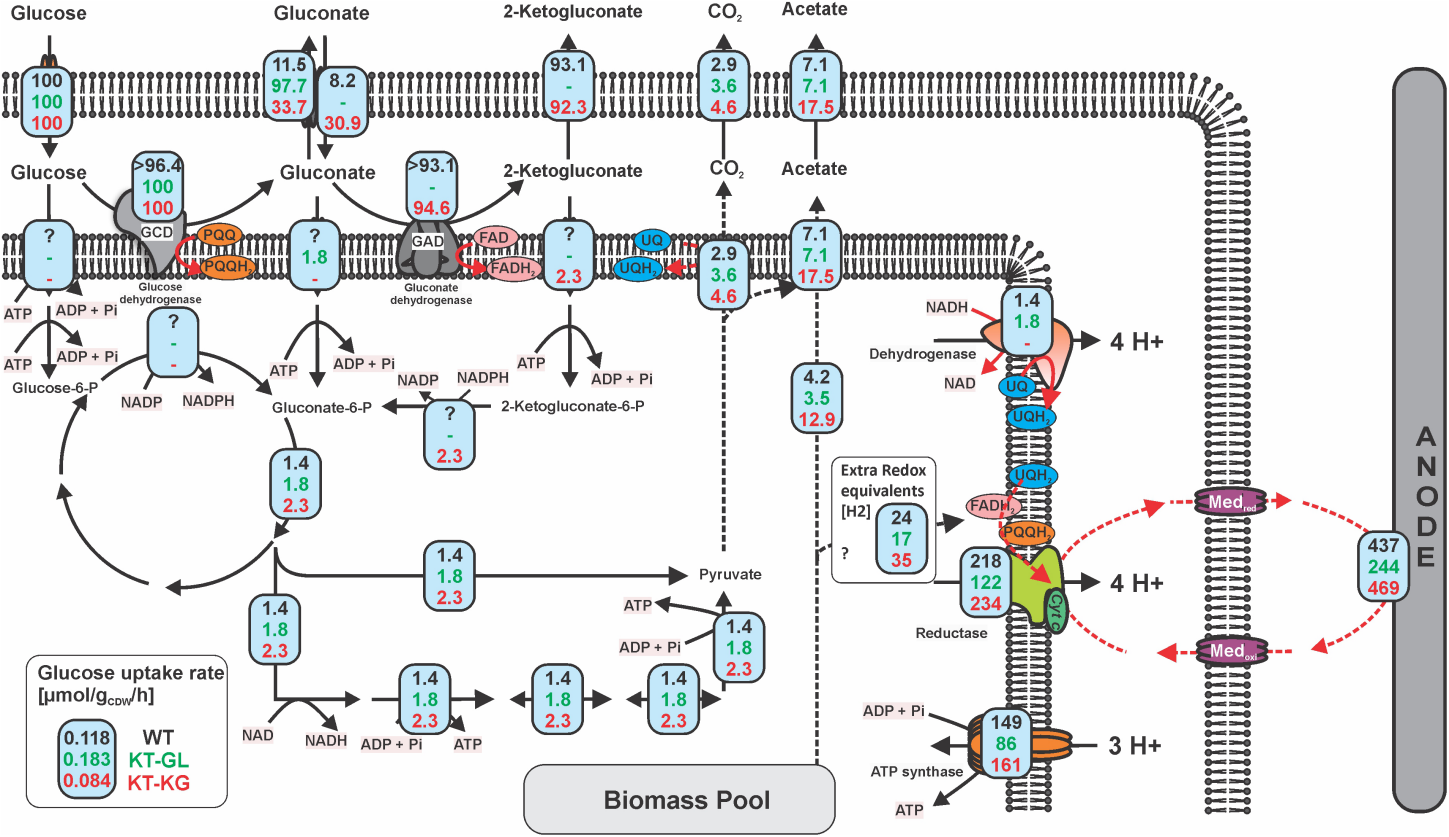
Flux balance analysis maps for the *P. putida* KT2440 wild type (black number), KT‐GL (green number) and KT‐KG (red numbers) strains in BES. Fluxes are normalized to the glucose uptake rate given in [mmol/g_CDW_/h] and calculated based on Supplementary Table S2. The fluxes through acetate and CO_2_ were estimated based on the ^13^C enrichment and on the assumption that cytoplasmic oxidation of sugar to acetate will release stoichiometric amounts of CO_2_, while degradation of storage carbon (e.g. poly‐hydroxybutyric acid) might not. The ATP rate was calculated based on the stoichiometric balance, considering the proton pumping effect of dehydrogenase and membrane reductase. In addition, the charge balance that one electron transferred to the anode would take away one proton from the periplasm to the extracellular space. Three protons are assumed to be needed for the synthesis of one ATP molecule. The ‘?’ in the flux indicates the uncertainties of the flux balance analysis, as it was unable to resolve the exact cross‐membrane flux for the WT. Here we assumed it followed the periplasmic oxidative pathway and then cytosolic glycolysis. The ‘‐’ means the respective pathway was metabolically not possible. ‘>’ means a minimum flux is given. Also, a mismatch between anodic electrons and predicted reductase activity (2 electron process) could point towards some redox equivalents originating from the biomass pool as well.

To further elucidate the carbon and electron fluxes in the different strains in BES, a flux balance analysis (FBA) was conducted, based on measured and calculated rates (see Supplementary Table S2) and the ratio of labelled vs non‐labelled carbon in acetate. Due to the extremely low metabolic turnover for the KT‐G strain, measurement uncertainties had a huge impact and the carbon balance remained below 10% when accounting for carbon partitioning in acetate. Therefore, the FBA was only performed for the WT, KT‐GL and KT‐KG strains (Figure 5).

The gene deletions only slightly altered the intracellular net fluxes. Despite the difference in terms of current densities, glucose consumption rates and acetate production yields stated above, the three strains maintained the intracellular carbon flux through the ED pathway to pyruvate at low levels (3.4, 6.5 and 3.9 μmol/g_CDW_/h for WT, KT‐GL and KT‐KG, respectively). Relative to the glucose uptake rate the highest glycolytic flux was observed in the KT‐KG strain (Figure 5). Acetate production rates ranged from ~8 to 14 μmol/g_CDW_/h and were strongly influenced by acetate coming from the unlabelled carbon pool (Biomass pool). In the KT‐KG strain, the relative contribution of acetate production from biomass reached almost 13% of the glucose uptake flux.

In a previous study, we showed that cytochrome C reductase is the likely site of electron withdrawal by the mediator in *P. putida* (Chukwubuikem et al., 2021; Lai et al., 2020). When balancing the redox equivalents (PQQH_2_, FADH_2_, UQH_2_ and NADH), we assumed that they enter the electron transfer chain at the usual sites and would contribute to the generation of a proton gradient accordingly. A further assumption considered charge balance. Per electron harvested via the anode, a positive charge has to be transferred to the cathode. We assume that these charges are protons since H_2_ formation is driven at the cathode. The number of protons translocated across the cytoplasmic membrane exceeds the charge transfer to the cathode. This means protons can drive the ATP synthase. Estimating the ATP synthase flux shows that the WT would have the highest absolute ATP flux available (47% better than the KT‐KG strain) but in relative terms, KT‐KG had the highest flux through the ATP synthase (Figure 5). For WT, KT‐GL and KT‐KG, the substrate‐level phosphorylation in lower glycolysis (at the level of phosphoglycerate and pyruvate kinase) will balance with sugar phosphorylation in the upper part of metabolism (at the level of glucokinase and phosphofructokinase), leaving only the ATP synthase as an additional source of ATP. In the KT‐G strain, the upper part needs 2 mol ATP per mol of Gluconate‐6P, which means that this route provides no net ATP gain. Compared to the lowest reported non‐growth associated maintenance in aerobic *P. putida* KT2440 at 920 μmol_ATP_/g_CDW_/h (Ebert et al., 2011) or even in the genome‐reduced *P. putida* KT2240 mutant at 710 μmol_ATP_/g_CDW_/h (Lieder et al., 2015), our maximum ATP generation of 147 μmol_ATP_/g_CDW_/h for the wild‐type, might explain why cells are barely surviving in the BES.

When matching the anode‐driven flux through the cytochrome C reductase with the fluxes producing redox equivalents a small deviation could be observed. The anode harvested experimentally more electrons than our FBA analysis suggests. This might indicate that the catabolism of biomass components towards acetate also released redox equivalents (Figure 5). This is experimentally supported by the observed currents in sugar‐free control experiments (Figure 3).

## DISCUSSION

In our previous work, we found that *P. putida* cells showed more oxidized redox carriers and a reduced ATP concentration, despite an increased adenylate energy charge when a current was produced. In addition, the cells only consumed glucose at a rate of 1%–3% compared to aerobic conditions and did not exhibit any growth (Yu et al., 2018). This active but extremely restricted phenotype sparked our interest in revealing metabolic constraints. We hypothesized that the route of glucose uptake might be a key determining factor in this process. To test this, we engineered three gene deletion mutants, that is KT‐G, KT‐GL and KT‐KG, capable of exclusively using one of the three described routes (Table 1). Since all three routes co‐exist in the wild‐type we could not expect that gene deletion would increase the overall performance, but inferior performance might indicate a limiting factor.

The KT‐GL mutant showed an almost identical aerobic growth profile as the WT. No significant difference was observed in terms of growth rate, glucose consumption and biomass yield (Table 2). This was well‐supported by literature showing that *P. putida* naturally had about 55%–78% of its total carbon flux via the gaT pathway (Kohlstedt & Wittmann, 2019; Nikel et al., 2015), while cultivated aerobically. Both KT‐G and KT‐KG exhibited a halved maximum growth rate, compared to the other two strains. The NADPH availability in KT‐KG should be lower than in the WT due to the involvement of 2K6PG reductase (KguD) and is assumed to be higher in KT‐G because of the enforced flux through glucose 6‐phosphate dehydrogenases (Zwf1‐3). The decreased growth rate for KT‐G was accompanied by a dramatically reduced glucose consumption rate and a slightly decreased biomass yield. However, the KT‐KG mutant consumed glucose at a slightly higher rate than the WT, but at a slightly lower biomass yield.

*P. putida* KT2440 wild‐type and mutants showed a distinctive phenotype in the BES compared to aerobic conditions. For instance, compared to the wild type, the glucose consumption rate was doubled in the mutant KT‐GL, while it was decreased in the KT‐KG mutant (Table 3). These results differed from those measured for aerobic cultivation (Table 2), as similar glucose consumption rates were measured for these three strains cultivated aerobically. In the BES, the secretion of gluconate as the end product increased the glucose turnover compared to the WT, while the secretion of 2‐ketogluconate as the end product showed the opposite effect.

The extremely low electrogenic activity of KT‐G in BES was unexpected. Our former studies using *P. putida* F1 or using fructose as the substrate revealed the intracellular redox equivalents were largely shifted to the oxidized pools (Lai, Yu, et al., 2016; Nguyen et al., 2021), and thus we hypothesized that the increased NADPH supply via the glcT pathway might play an important role in providing redox equivalents under BES conditions. Unfortunately, it was difficult to obtain reproducible data for the intracellular redox pools in our experimental set‐up, that would enable a direct assessment of the redox status of the cells. However, when looking at sugar consumption in KT‐G, we observed that it was almost eliminated with a specific consumption rate of only about 0.01 mmol/g_CDW_/h, which was less than 0.15% of the value cultivated aerobically. To double‐check that carbon reaches the cytoplasm, we conducted extra measurements of ^13^C labelling in the pyruvate molecule for the KT‐G strain (Supplementary Figure S9) in addition to acetate labelling (Figure 4). This confirmed that glucose‐derived carbon definitely reached the pyruvate node. We hypothesized that the increased ATP demand of the glcT pathway compared to the other two pathways could lead to energy starvation. The lower glycolytic reactions would just provide the needed amount of ATP to generate Gluconate‐6P, and in the absence of current, no ATP synthase activity could be expected unlike the other strains (Figure 5).

To further characterize this phenotype, we ran a BES experiment using the KT‐GL strain using gluconate as sole carbon source. In this situation, gluconate could only be taken up into cytosol but no periplasmic oxidation was possible, a comparable situation to the KT‐G strain on glucose, but with a difference in the NADPH balance and ATP demand of the upper pathway (Figure 5). The observed substrate consumption, current output and acetate enrichment showed very similar patterns to the KT‐G strain on glucose. This result supported the hypothesis, that periplasmic oxidation was needed for efficient current generation and carbon turnover.

However, pinpointing a cytosolic limitation purely on energy or redox requirements, remained elusive, since both experiments (KT‐G plus glucose, KT‐GL plus gluconate) gave comparable results at very different pathway stoichiometry. Despite different contributions from biomass carbon, the very similar accumulation of acetate in the three strains of *P. putida* KT2440 under BES conditions further made it difficult to attribute a limitation of the metabolism to redox balancing or energy demand. We can only hypothesize that regulatory mechanisms might contribute to the observed phenotype. Under aerobic conditions, a couple of transcriptional repressors (e.g. HexR, GnuR and PtxS) were reported in *P. putida* KT2440 to regulate the glucose metabolism (del Castillo et al., 2008), but if they play a role under anaerobic conditions in *P. putida* remains to be shown. Besides, a two‐component GtrS‐GltR system was reported in *Pseudomonas aeruginosa* that regulated glucose metabolism based on the presence of 2‐ketogluconate (Daddaoua et al., 2014). A similar mechanism (but acting on different key metabolites) might also exist in *P. putida* KT2440 regulating anaerobic cytosolic metabolism in BES. Our future work will focus on the one hand on obtaining redox cofactor measurements to finally shed light on the redox status in these strains and on the other hand on gene expression analysis to screen for a change in the expression of regulatory circuits under BES conditions.

In summary, we successfully generated strains that allowed us to study the glucose uptake routes. This showed that periplasmic oxidation to gluconate and 2‐ketogluconate are essential pathways for current generation in BES while direct uptake of glucose or gluconate was not supporting current formation. This indicated that cytosolic oxidation via the anode was constrained. We could also show the extent of the inoculated biomass contributing to the formation of acetate and, most likely, also to the anodic current. This new data shows that the cytosolic fluxes of glucose degradation were previously overestimated. Whether metabolism was constrained by a metabolic, a regulatory, or a combined mechanism remains to be studied.

## AUTHOR CONTRIBUTIONS

**Laura Pause:** Data curation (lead); formal analysis (lead); methodology (equal); visualization (supporting); writing – original draft (lead); writing – review and editing (equal). **Anna Weimer:** Data curation (supporting); formal analysis (lead); methodology (lead); writing – review and editing (equal). **Nicolas Wirth:** Conceptualization (supporting); methodology (lead); writing – original draft (supporting); writing – review and editing (equal). **Anh Vu Nguyen:** Data curation (supporting); writing – review and editing (equal). **Claudius Lenz:** Methodology (supporting). **Michael Kohlstedt:** Methodology (supporting); project administration (supporting); supervision (supporting); writing – review and editing (equal). **Christoph Wittmann:** Funding acquisition (lead); methodology (supporting); project administration (lead); resources (lead); supervision (lead); writing – review and editing (equal). **Pablo I. Nikel:** Conceptualization (lead); methodology (supporting); resources (lead); supervision (supporting); writing – review and editing (equal). **Bin Lai:** Conceptualization (lead); data curation (supporting); formal analysis (lead); funding acquisition (supporting); methodology (supporting); project administration (supporting); resources (supporting); supervision (lead); visualization (lead); writing – original draft (lead); writing – review and editing (lead). **Jens O. Krömer:** Conceptualization (lead); funding acquisition (lead); methodology (supporting); project administration (lead); resources (lead); supervision (lead); visualization (lead); writing – original draft (lead); writing – review and editing (equal).

## CONFLICT OF INTEREST STATEMENT

The authors declare no conflict of interest regarding the contents of this paper.

## Supporting information


Data S1.
