# Supplementary material for: Anaerobic glucose uptake in Pseudomonas putida KT2440 in a bioelectrochemical system
Source: Microb Biotechnol. 2023 Nov 22;17(1):e14375. doi: 10.1111/1751-7915.14375 (PMC10832537; doi:10.1111/1751-7915.14375)
Supplement: Supplementary file 1 — Data S1. [file MBT2-17-e14375-s001.docx]

**Supplementary materials**

***Anaerobic glucose uptake in Pseudomonas putida* KT2440 in a bioelectrochemical system**

Laura Pause^a^, Anna Weimer^b^, Nicolas T. Wirth^c^, Anh Vu Nguyen^a^, Claudius Lenz^a^, Michael Kohlstedt^b^, Christoph Wittmann^b^, Pablo I. Nikel^c^, Bin Lai ^d*^, Jens O. Krömer ^a^

^a^ Systems Biotechnology group, Helmholtz Centre for Environmental Research - UFZ, 04318 Leipzig, Germany

^b^ Institute of Systems Biotechnology, Saarland University, Campus A 1.5, 66123 Saarbrücken, Germany

^c^ Systems Environmental Microbiology Group, the Novo Nordisk Foundation Center for Biosustainability, Technical University of Denmark, 2800 Lyngby, Denmark

^d^ BMBF junior research group Biophotovoltaics, Helmholtz Centre for Environmental Research - UFZ, 04318 Leipzig, Germany

**Table of Contents**

| **Items** | **Descriptions** |
| --- | --- |
| Table S1 | Oligonucleotides used in this study. |
| Table S2 | Key process parameters of anaerobic glucose conversion of *P. putida* KT2440 mutants in BES. |
| Figure S1 | Growth profiles of the *P. putida* KT2440 wild type and the three gene deletion mutants. |
| Figure S2 | Semi-logarithmic plot of the growth profiles of *P. putida* KT2440 wild type and the three gene deletion mutants in DM9 medium transferred from a DM9 liquid preculture. |
| Figure S3 | Growth patterns of the wild type and gene deletion mutants of *P. putida* KT2440 determined using a high-throughput parallel cultivation system (BioLector, Beckman). |
| Figure S4 | Regression analysis for the determination of product/glucose yield coefficients for the strain *P. putida* KT2440 WT. |
| Figure S5 | Regression analysis for the determination of product/glucose yield coefficients for the strain KT-G. |
| Figure S6 | Regression analysis for the determination of product/glucose yield coefficients for the strain KT-GL. |
| Figure S7 | Regression analysis for the determination of product/glucose yield coefficients for the strain KT-KG. |
| Figure S8 | The secreted acetate concentrations of different strains in BES. |
| Figure S9 | The isotopic labelling in pyruvate produced by the KT-G strain in BES fed with ^12^C_6_-glucose or ^13^C_6_-glucose. |
| Figure S10 | BES fermentation of KT-GL strain with gluconate as the sole carbon source. |
|  |  |

Table S1. Oligonucleotides used in this study. Sequences used for the construction of the same plasmid via *USER* cloning are shaded in the same tone. Primer overhangs are separated from the annealing sequence by a space.

| **Name** | **Sequence (5’→3’)** | **Application** |
| --- | --- | --- |
| pSNW-USER_F | AGTCGACCUGCAGGCATGCAAGCTTCT | Linearization of suicide vector pSNW2 |
| pSNW-USER_R | AGGATCUAGAGGATCCCCGGGTACCG | Linearization of suicide vector pSNW2 |
| gad_HA1_fw | AGATCCU GCATGAGGTTGTCGTAGCC | Amplification of HA1 for the deletion of gad |
| gad_HA1_rv | ACGTCA CAUTGCGAATCCTCATCGGC | Amplification of HA1 for the deletion of gad |
| gad_HA2_fw | ATG TGACGUGTTTTTCAGCGCCC | Amplification of HA2 for the deletion of gad |
| gad_HA2_rv | AGGTCGACU CCATCTTGAGCAGGTCGTC | Amplification of HA2 for the deletion of gad |
| glk_HA1_fw | AGATCCU GAAAGTATCTGCCGTCGCC | Amplification of HA1 for the deletion of glk |
| glk_HA1_rv | A CTTCAUTTGAGGTGCTCCAGG | Amplification of HA1 for the deletion of glk |
| glk_HA2_fw | ATGAAG TGAGGGGGGCGCTTTGCG | Amplification of HA2 for the deletion of glk |
| glk_HA2_rv | AGGTCGACU CATCACGGTGGAACAGGCC | Amplification of HA2 for the deletion of glk |
| gtsABCD_HA1_fw | AGATCC UGTTGCACCTGGAAGCAAG | Amplification of HA1 for the deletion of gtsABCD |
| gtsABCD_HA1_rv | AGTCA CATCGGAGCACCTTTCTTGTTG | Amplification of HA1 for the deletion of gtsABCD |
| gtsABCD_HA2_fw | ATG TGACUCGTCTACACCATCAATA | Amplification of HA2 for the deletion of gtsABCD |
| gtsABCD_HA2_rv | AGGTCGACU CGCCGTCGAAGTACTTCTG | Amplification of HA2 for the deletion of gtsABCD |
| gnuK-gntT_HA1_fw | AGATCCU CATGACCTCGTGAATGGCC | Amplification of HA1 for the deletion of gnuK and gntT |
| gnuK-gntT_HA1_rv | A CATCAGTACCTCCTGCGG | Amplification of HA1 for the deletion of gnuK and gntT |
| gnuK-gntT_HA2_fw | ACTGATG UGACCGTTGATCGGGGTAATG | Amplification of HA2 for the deletion of gnuK and gntT |
| gnuK-gntT_HA2_rv | AGGTCGAC UGTCTGCAAGACCCTGGAC | Amplification of HA2 for the deletion of gnuK and gntT |
| PP_0652_HA1_fw | AGATC CUCACGGCACAACTCATCC | Amplification of HA1 for the deletion of PP_0652 |
| PP_0652_HA1_rv | AGTTCA AGTCATTCAAGGAGTCTCGGTT | Amplification of HA1 for the deletion of PP_0652 |
| PP_0652_HA2_fw | AC TTGAACUCGAGTCAACGGG | Amplification of HA1 for the deletion of PP_0652 |
| PP_0652_HA2_rv | AGGTCGACU GCCGAGTTCCTTTTCCTCG | Amplification of HA1 for the deletion of PP_0652 |

Table S2. Key process parameters of anaerobic glucose conversion of *P. putida* KT2440 mutants in BES. [Fe(CN)_6_]^3−/4−^ was used as the electron transfer mediator and the anode potential poised at +0.697 V vs SHE.

|  | **WT** | **KT-G*** | **KT-GL** | **KT-KG** |
| --- | --- | --- | --- | --- |
| CB [%] | 100.0 | 26.7 | 101.3 | 103.9 |
| EB [%] | 101.1 | 19.2 | 102.2 | 104.8 |
| **Yield [mmol/mmol_glucose]** | | | | |
| Y2kga/glc | -0.93 ± 0.04 |  |  | -0.92 ± 0.04 |
| Yga/glc | 0.08 ± 0.04  -0.12 ± 0.03 |  | -0.98 ± 0.05 | 0.31 ± 0.12  -0.34 ± 0.04 |
| Yace/glc | -0.07 ± 0.01 | -0.48 ± 0.06 | -0.07 ± 0.01 | -0.18 ± 0.01 |
| Yco2/glc ** | -0.07 ± 0.01 | -0.48 ± 0.06 | -0.07 ± 0.01 | -0.18 ± 0.01 |
| Ypyr/glc |  | -0.06 ± 0.02 |  |  |
| Yelectrons/glc | -4.37 ± 0.08 | -0.22 ± 0.17 | -2.44 ± 0.12 | -4.69 ± 0.10 |
| **Rate [mmol/g_CDW_/h]** | | | | |
| r_glc_ | -0.12 ± 0.02 | -0.01 ± 0.01 | -0.18 ± 0.01 | -0.08 ± 0.01 |
| r_2kga_ | 0.11 ± 0.02 |  |  | 0.08 ± 0.01 |
| r_ga_ | -0.01 ± 0.01  0.01 ± 0.01 |  | 0.18 ± 0.01 | -0.03 ± 0.01  0.03 ± 0.01 |
| r_ace_ | 0.01 ± 0.01 | -0.01 ± 0.01 | 0.01 ± 0.01 | 0.02 ± 0.01 |
| r_pyr_ |  | -0.0007 ± 0.0003 |  |  |
| r_electrons_ | 0.51 ± 0.10 | 0.003 ± 0.002 | 0.45 ± 0.01 | 0.39 ± 0.03 |

*Note: The extremely low turnover rate for KT-G strain makes the systematic error (e.g. analytic resolution, etc) plays a determining role in these values.

** the CO_2_ yield was estimated based on the biochemical coefficient that one acetate would correspond to one CO_2_ release.

The yields were calculated using the linear regression functions as shown in the figure S4-S7 below. The glucose consumption rate was calculated by plotting the glucose quantity against the time normalized to the integrated averaged biomass quantity over the batches. The rates of the metabolic products and electrons were then calculated by the equation: r_product_ = r_glucose_ * Y_product/glucose_ [mmol_product/gCDW/h].

The carbon balance and electron balance were calculated used the equations below:

$$CB \left[ \% \right]= \left( Y_{2kga}*6+ Y_{ga}*6+ Y_{ace}*2+ Y_{CO2}*1 \right)/6*100$$

$$EB \left[ \% \right]= \left( Y_{2kga}*6*{DoR}_{2kga}+ Y_{ga}*6* {DoR}_{2kga}+ Y_{ace}*2* {DoR}_{2kga}+ Y_{electrons} \right)/{(6}*{DoR}_{glc})*100$$

DoR: degree of reduction, calculated for chemical C_a_H_b_O_c_N_d_ as: DoR = (a*4 + b*1 + c*(-2) + d*(-3))/a.

The numbers in the CB and ED equations are the carbon atom number in the respective chemicals.


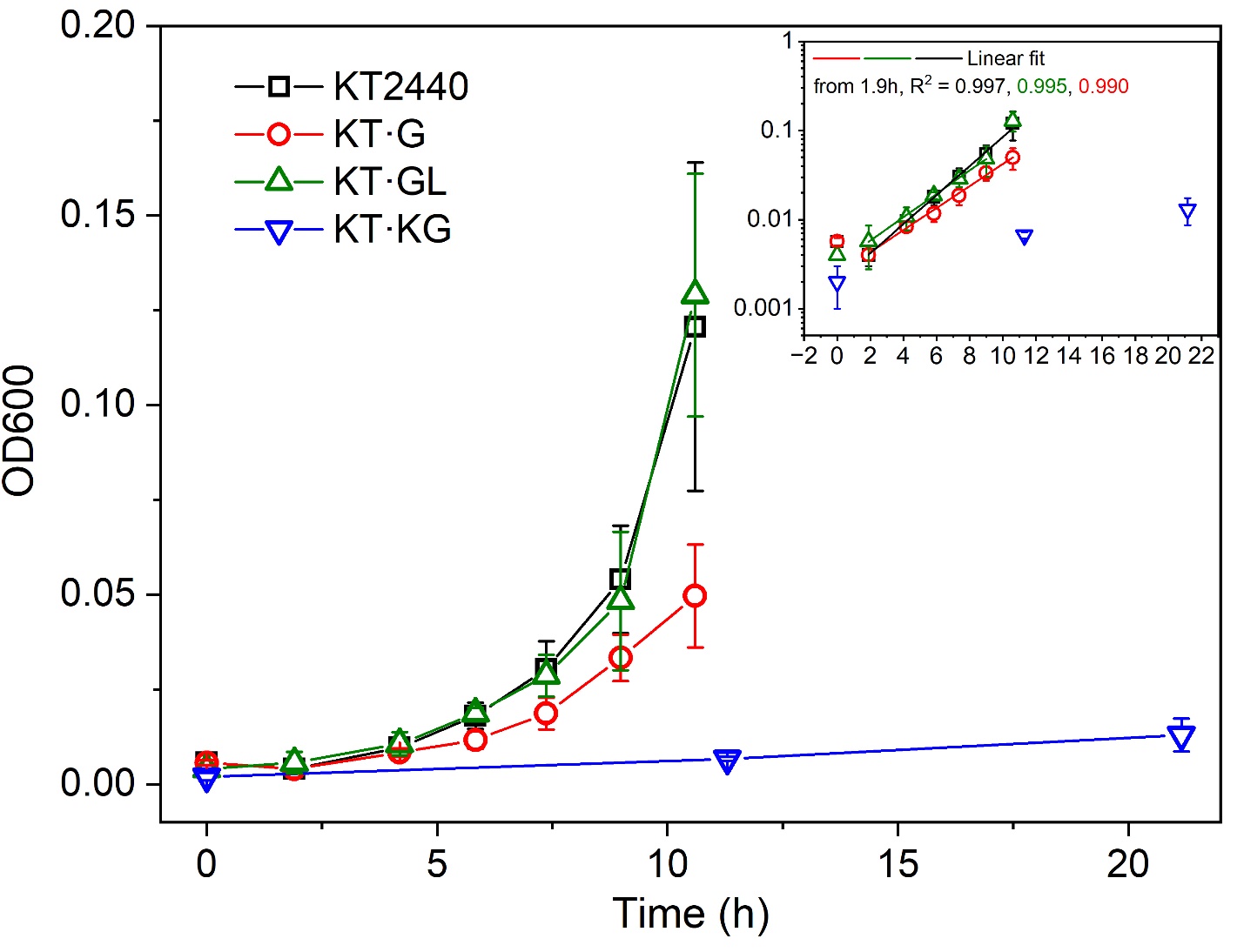
**Figure S1. Growth profiles of the *P. putida* KT2440 wild type and the three gene deletion mutants in DM9 medium directly from LB plate culture**. All the strains were reactivated from cryo-stocks on LB agar plate and then the colonies were picked up and inoculated into DM9 medium with 5g/L glucose. Each strain was done with 3 biological replicates.


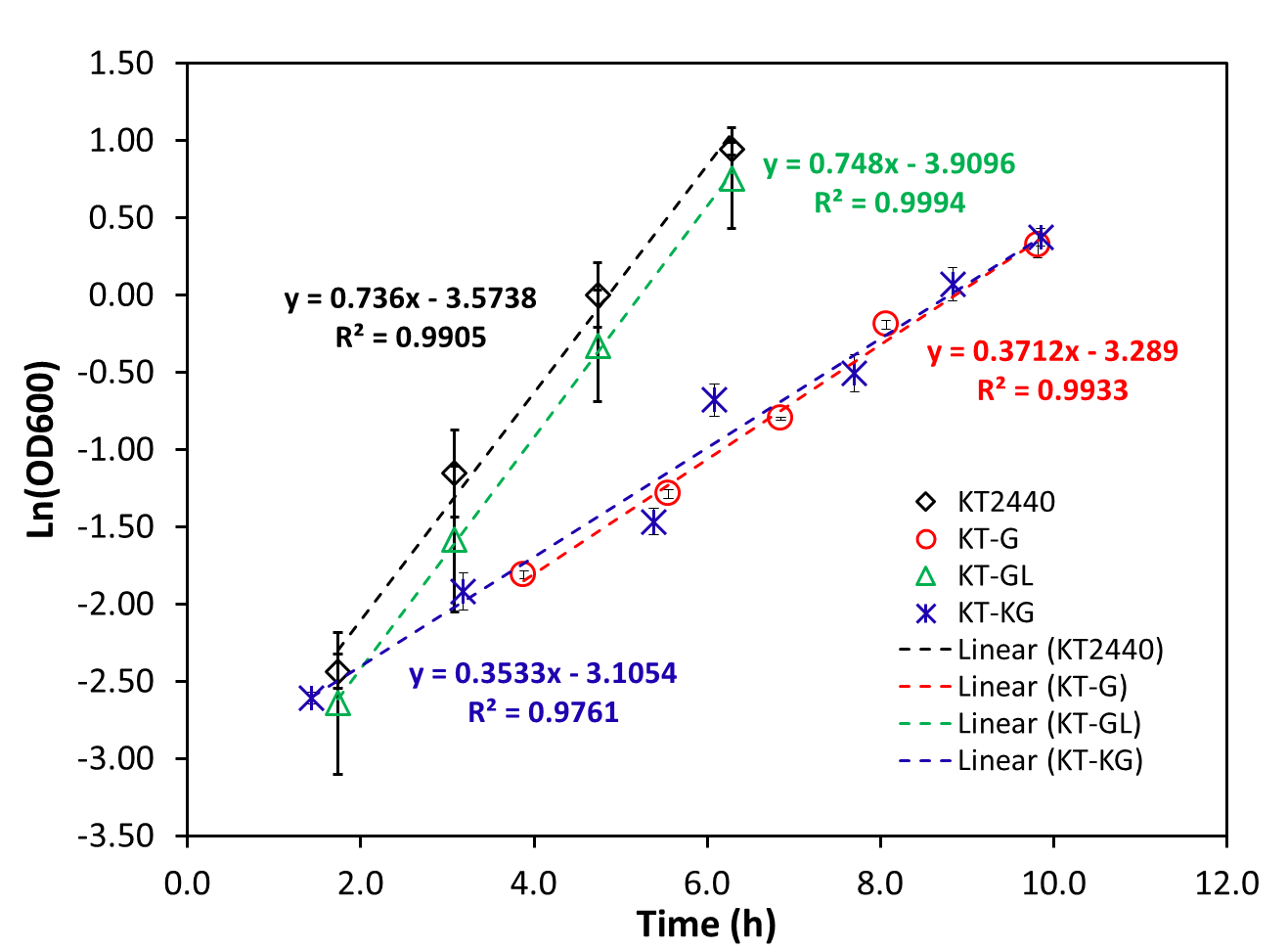


**Figure S2 Semi-logarithmic plot of the growth profiles of *P. putida* KT2440 wild type and the three gene deletion mutants in DM9 medium transferred from a DM9 liquid preculture.** This figure refers the growth data presented in the main manuscript Figure 2 (the subplot A). The linear range indicates exponential growth, the slope represents the maximal growth rate. The single colony was picked up from the LB plate and then inoculated into fresh DM9 medium. After over night growth, the obtained DM9 culture was used as preculture to inoculate further flasks with fresh DM9 medium to determine the growth kinetics stated in this figure. Three biological replicates were used.


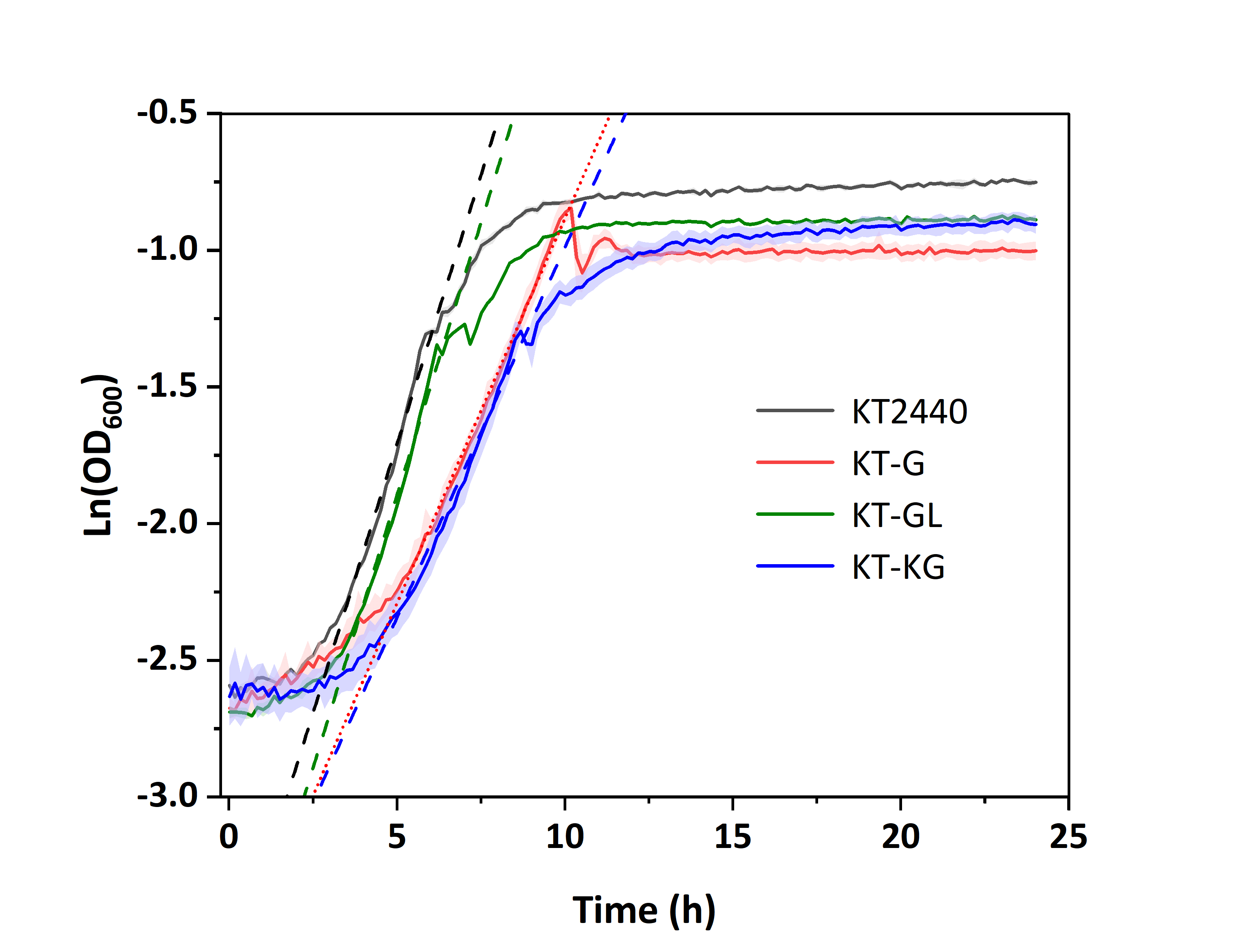


**Figure S3 Growth patterns of the wild type and gene deletion mutants of *P. putida* KT2440 determined using a high-throughput parallel cultivation system (BioLector, Beckman).** Precultures were prepared as for the growth test for Figure 2A and Figure S2 (i.e. Single colony on LB plate -> DM9 liquid culture -> BioLector cultivation) . Each well contained 1000 µL DM9 medium with 5 g/L glucose. Each strain was done with 3 biological replicates, and the lines show the average data.


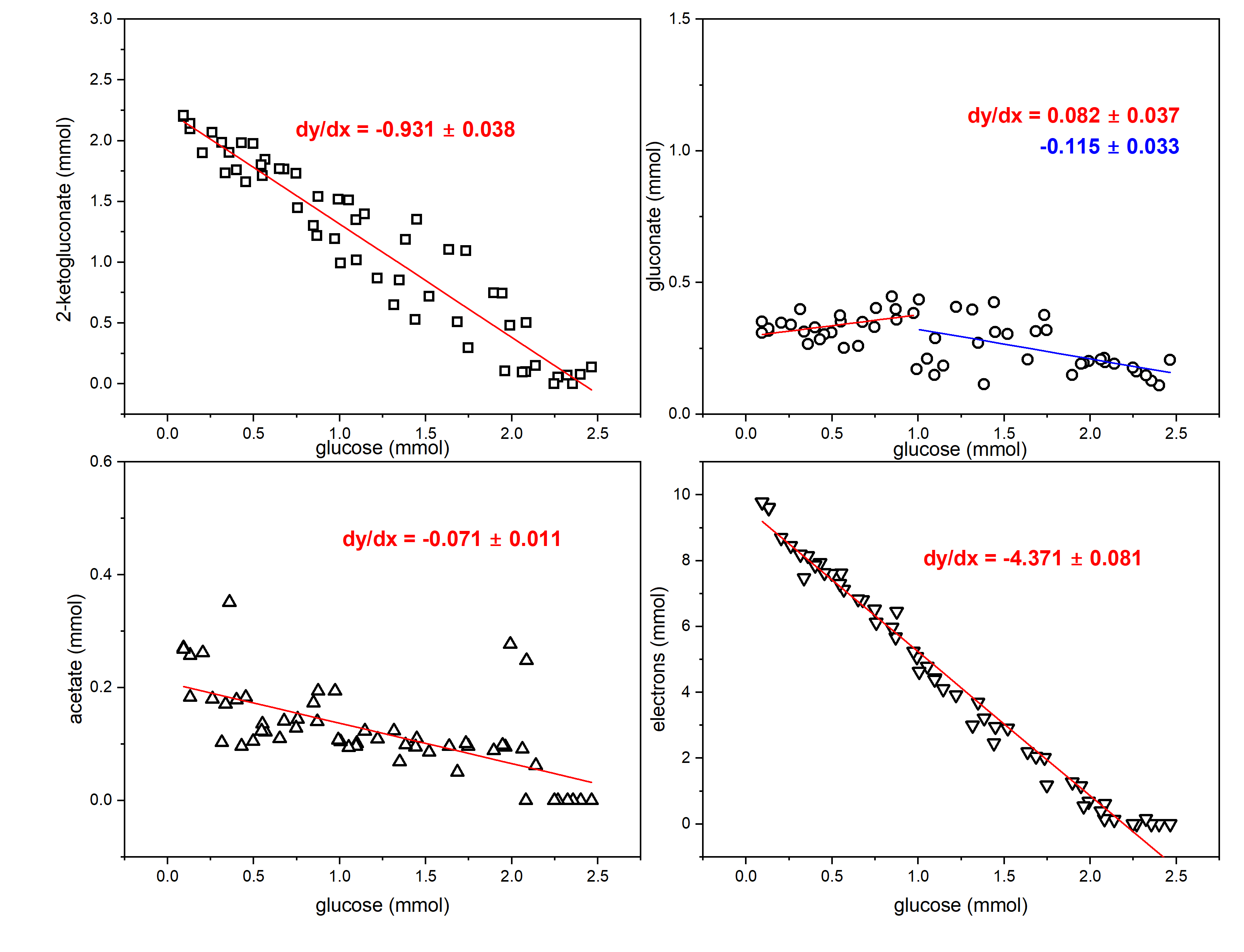


**Figure S4. Regression analysis for the determination of product/glucose yield coefficients for the strain *P. putida* KT2440 WT**. The analysis was done using OriginPro 2022b with 55 data points from 6 biological replicates.


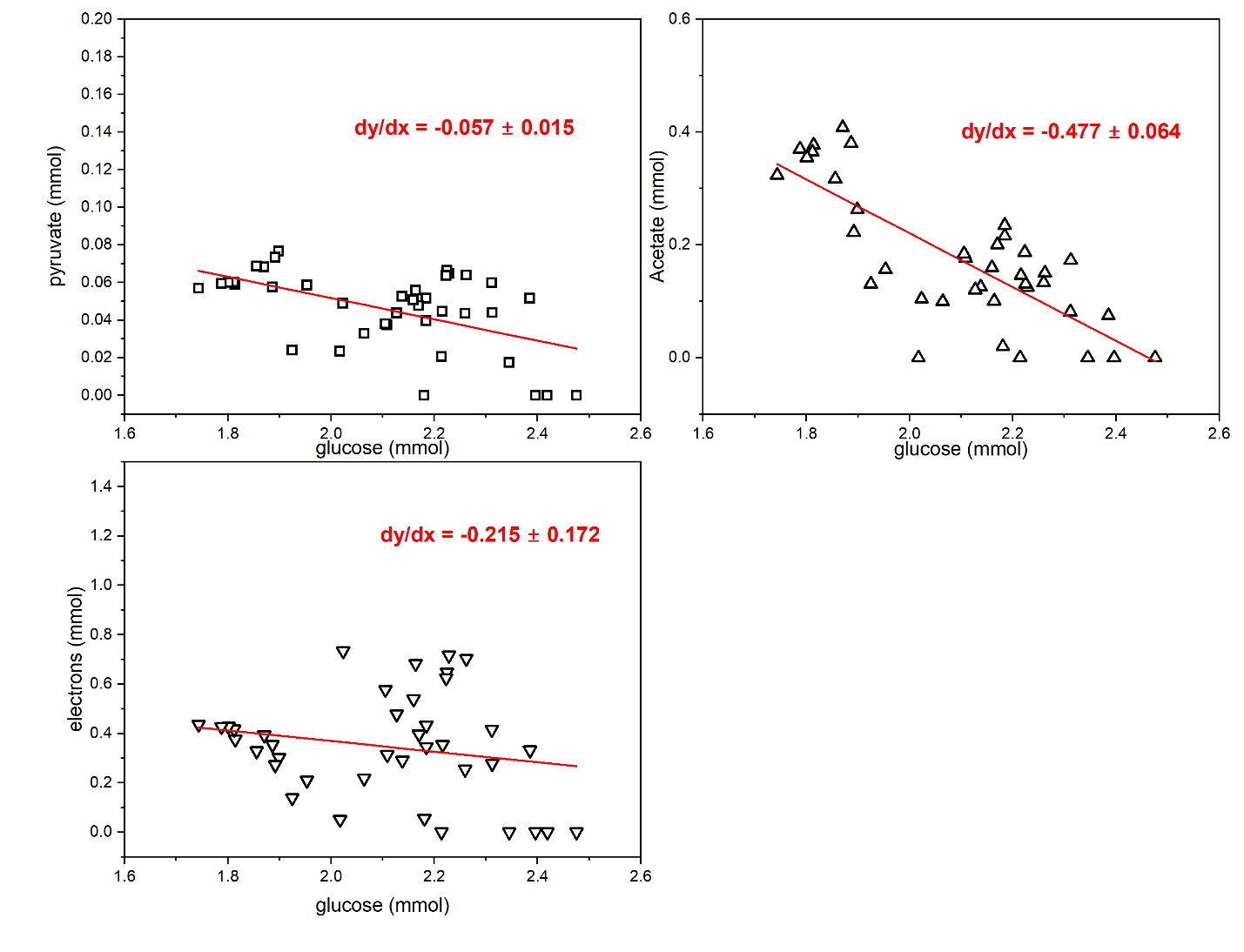


**Figure S5. Regression analysis for the determination of product/glucose yield coefficients for the strain KT-G**. The analysis was done using OriginPro 2022b with 51 data points from 6 biological replicates (except 37 data points from 4 biological replicates).


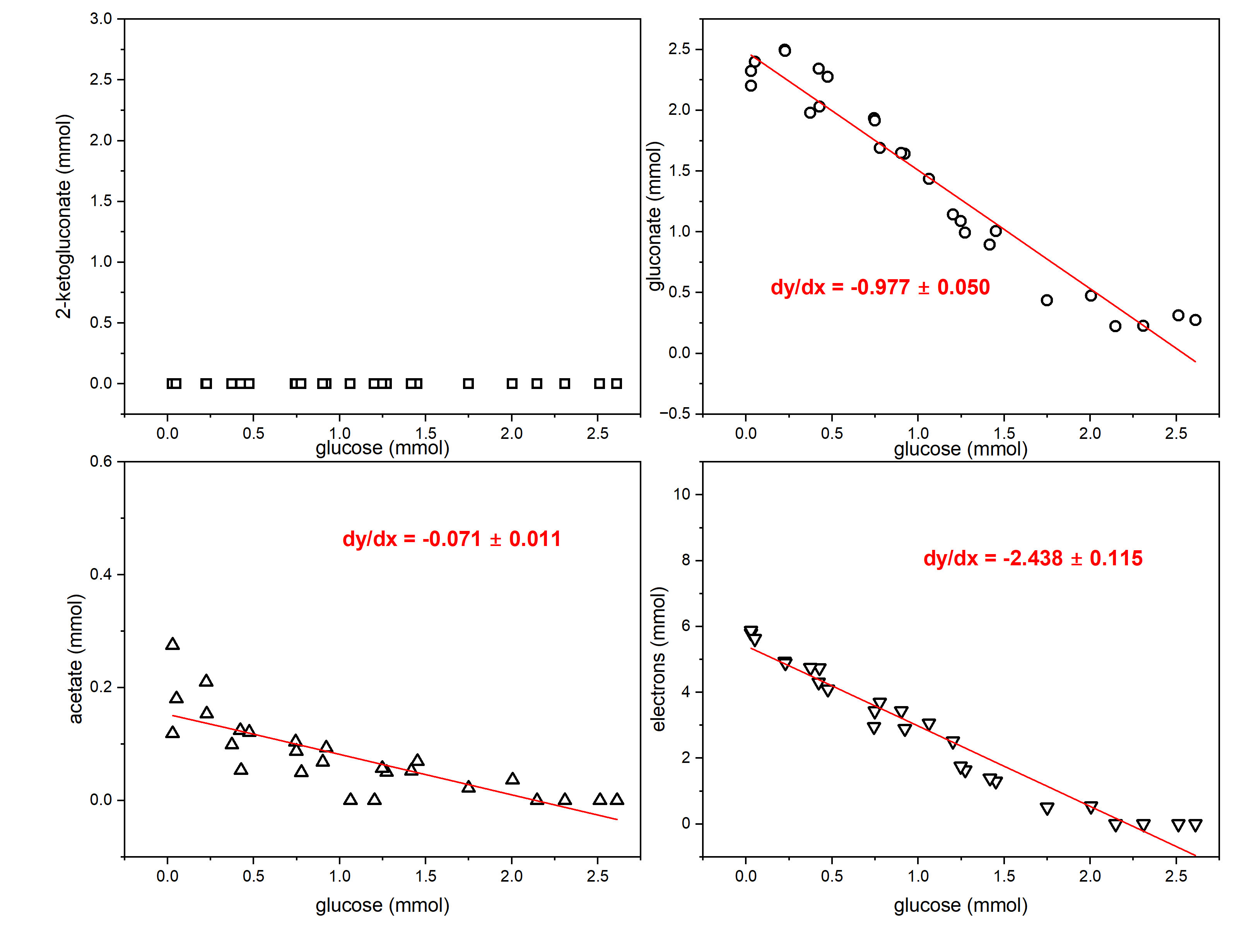


**Figure S6. Regression analysis for the determination of product/glucose yield coefficients for the strain KT-GL**. The analysis was done using OriginPro 2022b with 26 data points from 4 biological replicates.


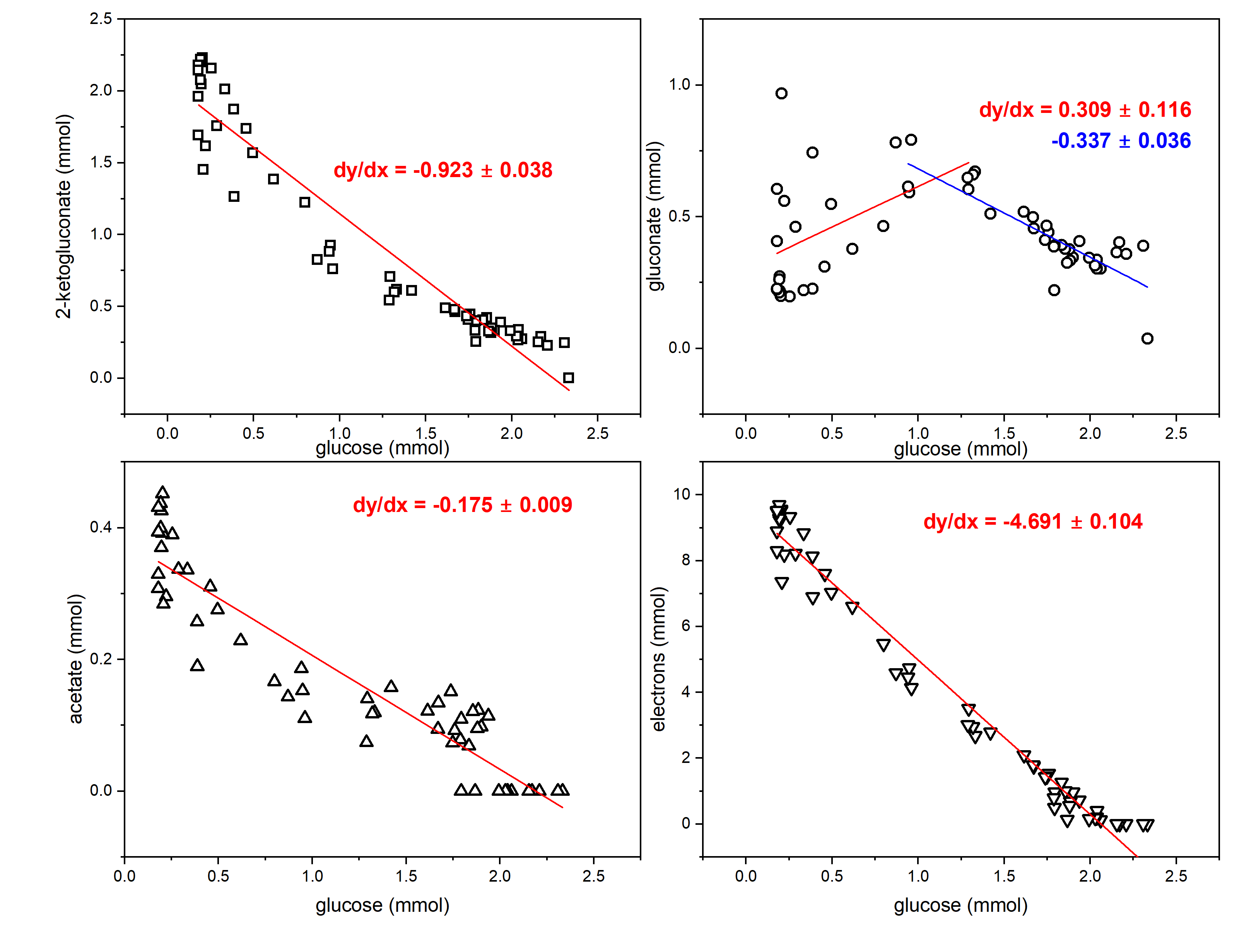


**Figure S7. Regression analysis for the determination of product/glucose yield coefficients for the strain KT-KG**. The analysis was done using OriginPro 2022b with 56 data points from 6 biological replicates.





**Figure S8. The secreted acetate concentrations of different strains in BES**. The data points present the acetate concentrations during the fermentation batches from inoculation to the end of the batch. KT-GL (gluconate): BES fermentation of the KT-GL strain with gluconate as the sole carbon source. KT-G (none): KT-G strain without added carbon source. The released acetate represents basal acetate formation from biomass components.


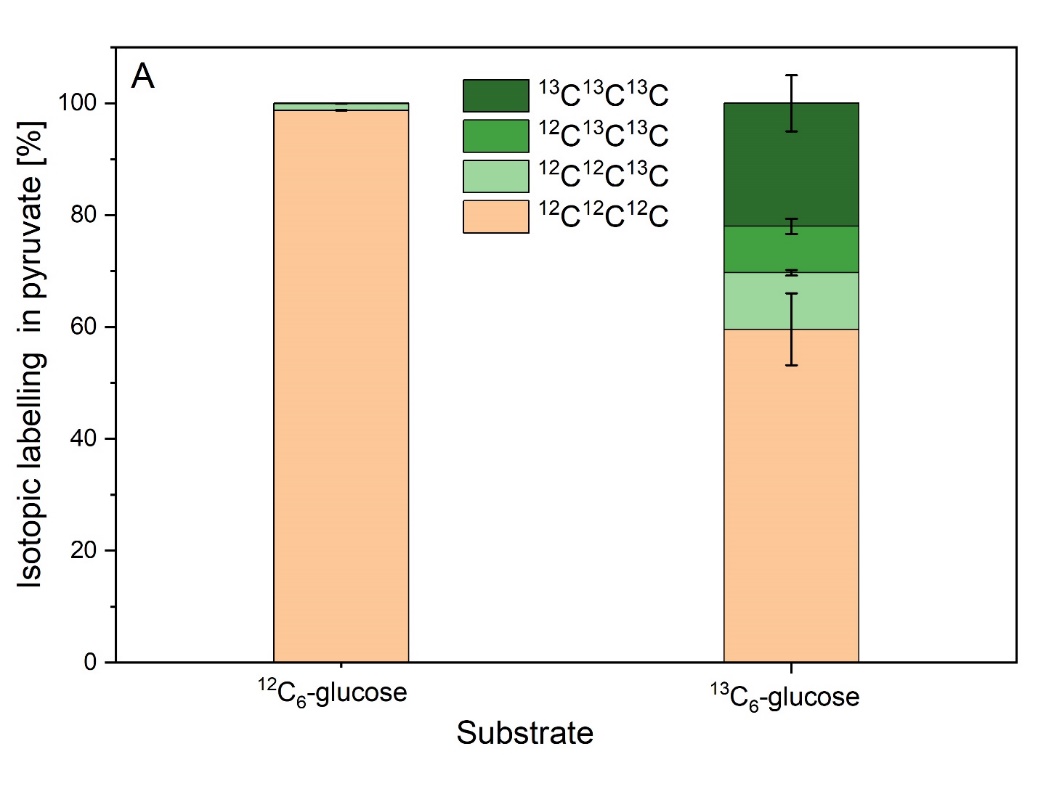


**Figure S9. The isotopic labelling in pyruvate produced by the KT-G strain in BES fed with ^12^C_6_-glucose or ^13^C_6_-glucose.**  The error bar represents the deviation of 3 biological replicates.

The mass isotopomer distribution of pyruvate was determined using gas chromatography coupled to mass spectrometry (GC-MS). Briefly, 200µL of fermentation supernatant were dried under nitrogen stream. Derivatization was done in a two-step procedure: First, dried samples were dissolved in 50µL methoxyamine hydrochloride in pyridine (20mg/mL), and incubated at 80 °C for 30 min. Afterwards, 50µL MSTFA (Macherey-Nagel, Düren, Germany) were added, and incubated likewise. Mass fragment distribution of pyruvate was determined by GC/MS (Agilent 7890A, Quadrupole Mass Selective Detector 5975C, Agilent Technologies) using the following oven program: 30 °C (0-1 min), 10 °C min^−1^ increase (1-10 min), and 40 °C min^−1^ increase (10-15.125 min). Selected ion monitoring (SIM) targeting m/z 174 was performed to quantify the mass isotopomer fractions *m+0*, *m+1*, *m+2*, *m+3* of a fragment ion containing all carbon atoms of pyruvate. The obtained ^13^C enrichment was corrected for natural abundance of stable isotopes^[[1]](#footnote-1)^, and was expressed as summed fractional labelling^[[2]](#footnote-2)^.





**Figure S10. BES fermentation of KT-GL strain with gluconate as the sole carbon source**. A) fermentation profile. B) the isotopic analysis of secreted acetate in the BES medium. Error bar represents the deviation of biological replicates.

Labeled gluconate was not available for this experiment. Instead, the preculture of KT-GL was prepared in shake-flasks using ^13^C_6_-glucose as the substrate. After overnight cultivation, the fully labeled biomass was harvested and inoculated into BES reactors with unlabelled gluconate as the sole carbon source. This yields an inverse labeling ratio compared to the situation with unlabeled biomass and ^13^C-glucose as substrate. Here, the fraction of ^12^C indicates the carbon originating from the carbon source in the BES reactor, while the ^13^C shows the biomass fraction of acetate.

1. van Winden, W.A., Wittmann, C., Heinzle, E., and Heijnen, J.J. (2002) Correcting mass isotopomer distributions for naturally occurring isotopes, Biotechnol. Bioeng. 80: 477-479. [↑](#footnote-ref-1)
2. Wittmann, C., and Heinzle, E. (2005) Metabolic activity profiling by 13C tracer experiments and mass spectrometry in Corynebacterium glutamicum. In: Microbial Processes and Products. Barredo, J.-L. (ed). Totowa, NJ: Humana Press. 191-204. [↑](#footnote-ref-2)
